# Supplementary material for: Chronic kidney disease and associated factors among adult population in Southwest Ethiopia
Source: PLoS One. 2022 Mar 3;17(3):e0264611. doi: 10.1371/journal.pone.0264611 (PMC8893675; doi:10.1371/journal.pone.0264611)
Supplement: S1 Questionnaire — (PDF) [file pone.0264611.s001.pdf]

English version of questionnaire

Dear participant!

My name is _______________ currently we are working at College of Health Sciences, Mizan Tepi University. We are conducting a survey on Chronic kidney disease and associated factors among adult population in Southwest Ethiopia.

The ultimate purpose of this survey is to collect information necessary for developing programs to prevent chronic disease especially chronic kidney disease and its sequels. To attain this purpose your honest and genuine participation is very important and highly appreciable. We, therefore, kindly request you to give as accurately and carefully as possible information during the interview. Please be assured that all the information gathered will be kept strictly confidential and you do not need to give your name or any special identification that might disclose who you are. Only the researcher has the access of the information and used it for the study purpose only.

You have a full right to interrupt or not to participate in this study.

Data Collector

Name __________________________________ Signature ______________ Date _________

Supervisor

Name __________________________________ Signature ______________ Date __________

Consent form

In signing this document, I am giving my consent to participate in the study entitled "Chronic kidney disease and associated factors among adult population in Southwest, Ethiopia."

I have been informed that the purpose of this research project and I understand that I am selected to participate in this study randomly. I have been informed that my participation in this study is willing full and voluntary even I have right to refuse or interrupt the interview and my name will not be mentioned on the questionnaire.

I, undersigned, have understood the purpose of the study & fully agree to participate in the study.

Signature of the participant------------------- Date -------------------------

Thank you!!!

District/town: ____________
Kebele:_________________
Village: _________________
House number (code)_______

Socio-demographic and economic characteristics

No	Questions	Options	Skip
101	Gender	1. Male<br>2. Female	
102	How old are you?	Age in years_________	
103	What is your Educational level?	1. Who can't read and write?<br>2. Who can read and write?<br>3. Primary school (1-8th Grade)<br>4. Secondary school (9th-12th Grade)<br>5. Higher education	
104	What is your current Marital status?	1. Single<br>2. Married<br>3. Divorced<br>4. Widowed	
105	What is your main Occupation?	1. Gov employee<br>2. Non gov. employee<br>3. Student<br>4. Housewife<br>5. House maid<br>6. Retired<br>7. Merchant<br>8. Others specify	
106	What is your Religion?	1. Orthodox<br>2. Muslim<br>3. Catholic<br>4. Protestant<br>5. Other, specify	
107	What is your ethnicity?	1. Kefa<br>2. Bench<br>3. Amahara<br>4. Tigray<br>5. Oromo<br>6. Cheka<br>7. Other____________ (specify) ________________	
108	What is the total monthly income of your household? (From all sources and all members. Please ask income in kind and cash. If in kind use the current market price to convert it to cash income)	Enter the No. in Ethiopian birr (ETB)_______________	

109	Where is the main source of water for drinking?	1. Pipe<br>2. Spring<br>3. Wall<br>4. Other (specify)	
110	How many litters of water you drink per day?	-------------litters per day	
**Behavioral or lifestyle characteristics**			
**Alcohol and cigarette consumption related questions**			
111	Have ever smoke cigarette?	1. Yes<br>2. No	2 to 114
112	For how many years have you smoked?	--------years	
113	How many cigarettes do you smoke per day?	----------cigarettes per day	
114	Have ever drank alcohol?	1. Yes<br>2. No	2 to 117
115	For how many years have you drank?	--------years	
116	How many litters of alcohol drink per day	-------------litters per day	
117	Do you add salt to your food?	1. No<br>2. Occasionally<br>3. Often<br>4. With each meal	
118	Do you eat canned or processed food?	1. No<br>2. Occasionally<br>3. Few times a week<br>4. Every day	
**Physical activity related questions**			
119	Does your work involve vigorous-intensity activity that causes large increases in breathing or heart rate like [carrying or lifting heavy loads, digging or construction work] for at least 10 minutes continuously	1. yes<br>2. no	2 to 122
120	In a typical week, on how many days do you do vigorous-intensity activities as part of your work?	--------number of days	2 to 125
121	How much time do you spend doing vigorous-	hours : minute<br>└─┴─┘ : └─┴─┘	

	intensity activities at work on a typical day?		
122	Does your work involve moderate-intensity activity that causes small increases in breathing or heart rate such as brisk walking [or carrying light loads] for at least 10 minutes continuously?	1. yes<br>2. no	
123	In a typical week, on how many days do you do moderate-intensity activities as part of your work?	--------number of days	
124	How much time do you spend doing moderate-intensity activities at work on a typical day?	hours : minute<br>└─┴─┘ : └─┴─┘	
**Recreation / exercise related questions**			
125	Do you engage in exercises that involve vigorous intensity (causes large increases in breathing or heart rate for at least 10 minutes continuously)		2 to 128
126	In a typical week, on how many days do you do vigorous-intensity exercise as part of your work?	-------number of days	
127	How much time do you spend doing vigorous-intensity exercise at work on a typical day?	hours : minute<br>└─┴─┘ : └─┴─┘	
128	Does your exercise involve moderate-intensity exercise that causes small increases in breathing or heart rate at least 10 minutes continuously?	1. yes<br>2. no	2 to 131
129	In a typical week, on how many days do you do moderate-intensity exercise as part of your work?	-----number of days	
130	How much time do you spend doing moderate-	hours : minute<br>└─┴─┘ : └─┴─┘	

	intensity exercise at work on a typical day?		
**History High blood pressure**			
131	Do you have high blood pressure or take medicine for high blood pressure?	1. Yes<br>2. No	2 to 133
132	How long ago you were first diagnosed?	1. < 1 year<br>2. [1-3 years<br>3. [3-5 years<br>4. [5-10 years<br>5. ≥ 10 years	
133	Do you check your blood pressure at home?	1. Yes<br>2. No	2 to 136
134	If yes, how often?	1. Daily<br>2. Several times per week<br>3. Once per week<br>4. Once per month)	
135	How often is your blood pressure greater than 140/90?	1. Most of the time<br>2. Occasionally<br>3. Never	
136	Do you exercise to reduce BP?	1. Yes<br>2. No	2 to 138
137	If you exercise, how often?	1. At least daily<br>2. 3 times per week<br>3. Once a week<br>4. Once a month	
**Cardiovascular/heart diseases related questions**			
138	Have you had a stroke? Y	1. Yes<br>2. No	
139	Do you have heart failure?	1. Yes<br>2. No	
140	Have you had a heart attack?	1. Yes<br>2. No	
141	Have you had a surgery for arteries supplying the legs?	1. Yes<br>2. No	
**Kidney Disease related questions**			
142	Have you ever been told you have kidney disease?	1. Yes<br>2. No	2 to 147
143	How long has it been since you were first diagnosed?	1. < 1 year<br>2. [1-3 years<br>3. [3-5 years<br>4. [5-10 years<br>5. ≥10 years	
144	Have you been told what caused your kidney disease	1. Yes<br>2. No	

145	What caused your kidney disease?	1. Diabetes<br>2. High blood pressure<br>3. Glomerulonephritis<br>4. Kidney stones<br>5. Medication<br>6. surgery<br>7. severe medical illness<br>8. Others	
146	Have you ever had any of the following?<br>More than one answer is possible	1. Kidney problems at birth or in childhood?<br>2. Hospitalization due to kidney failure?<br>3. Kidney failure while hospitalized for another reason?<br>4. Kidney stones?<br>5. Bladder or kidney infections?<br>6. Difficulty emptying your bladder?<br>7. Bladder or other urologic surgery?<br>8. Radiation to the abdomen or pelvis?<br>9. Chemotherapy for cancer?<br>10. Family history of kidney disease?<br>11. Blood in the urine?	
**Medications**			
147	Do you use regularly pain or anti-inflammatory medicines or NSAIDS (i.e. Aleve, naproxen, ibuprofen, Motrin)?	1. Yes<br>2. No	2 to 149
148	If yes, how often?	1. At Least Daily<br>2. 3 Times Per Week<br>3. Once A Week<br>4. Once A Month	
149	Do you use herbal supplements?	1. Yes<br>2. No	2 to 151
150	If yes, list them	------------------------	
**Diabetes**			
151	Have you ever been told you have diabetes?	1. Yes<br>2. No	2 to 160
152	How long ago were you first diagnosed?	1. < 1 year<br>2. [1-5) years<br>3. [5-10) years<br>4. ≥10 years	

153	Have you ever taken pills for diabetes?	1. Yes<br>2. No	
154	If yes, how many years did you take it?	1. < 1<br>2. [1-5) years<br>3. [5-10) years<br>4. > 10	
155	Are currently taking insulin/pills for diabetes?	1. Yes<br>2. No	
156	If you have stopped taking, how long ago did you stop (years)?	1. < 1 years<br>2. [1-5 years<br>3. [5-10 years<br>4. ≥ 10 years	
157	How well have you blood sugars been controlled? Usually	1. < 100<br>2. [100-150<br>3. [150-200<br>4. >200<br>5. I don't check them	
158	Do you have eye disease from diabetes?	1. Yes<br>2. No	
159	Have you had laser treatment for your eyes?	1. Yes<br>2. No	
**Anemia**			
160	Have you ever been told you were anemic, had a low blood or hemoglobin count?	1. Yes<br>2. No	2 to 164
161	How long ago you were first diagnosed?	1. < 1 year<br>2. [1-3 years<br>3. [3-5 years<br>4. [5-10 years<br>5. >10 years	
162	Have you had to take medication to prevent anemia?	1. Yes<br>2. No	
163	If yes what type:	Folate or folic acid: Dose: ____<br>Iron (pills or injections): Dose: __<br>Vitamin B12: Dose: _____<br>Epogen or Aranesp: Dose: _	
164	Do you have any black stools?	1. Yes<br>2. No	
165	Do you have any bright red blood in your stool?	1. Yes<br>2. No	
166	Do you have any blood in your urine?	1. Yes<br>2. No	
167	Do you have a family history of anemia?	1. Yes<br>2. No	

168	If female, do you still menstruate?	1. Yes<br>2. No	2 to 170
169	If yes, how often:	-----------------	
170	Have you ever been diagnosed with the following	1. Lymphoma<br>2. Leukemia<br>3. Vomiting blood<br>4. Stomach ulcers<br>5. Recurrent nosebleeds	

Physical measurements

171	Height	In cm______________
172	Weight	In kg ______________
173	Waist circumference	In cm______________
174	Blood Pressure	
	Reading1	Systolic(mmHg) --------
		Diastolic (mmHg)-------
	Reading 2	Systolic(mmHg) --------
		Diastolic (mmHg)-------

Laboratory measurements

No	To be measured	Options	Remark
175	Proteinuria	1. Positive<br>2. Negative	
176	Hematuria	1. Positive<br>2. Negative	
177	Blood glucose level	---------------mg/dl	
178	Creatinine level	---------------mg/ml	
179	GFR	-------------ml/min/1.73m^2	

Amharic version of questionnaire (local language)

ወረዳ:_________________

ቀበሌ:_________________

መንደር:_________________

የቤት ቁጥር(ኮድ)____________

ክፍል 1: አጠቃላይ መረጃ

ተ.ቁ	ጥያቄ	መልስ	Skip
101	ጾታ	1. ወንድ<br>2. ሴት	
102	ዕድሜ	______________________አመት	
103	የትምህርት ደረጃዎ ስንት ነዉ ?	1. ማንበብም-ሆነ-መጻፍ አልችልም<br>2. ማንበብ ና መጻፍ ብቻ እችላለሁ<br>3. የመጀመሪያ ደረጃ(1-8)<br>4. ሁለተኛ ደረጃ(9-12)<br>5. ኮሌጅና ከዚያ በላይ(12+)	
104	የጋብቻ ሁኔታ	1. ያገባ<br>2. ያላገባ<br>3. የፈታ/የፈታች<br>4. ባል/ሚስት የሞተባት/የሞተበት	
105	ዋና የስራ ዘርፍዎ ምንድ ነዉ?	1. ገበሬ/አርሶ አደር<br>2. የመንግስት ስራ<br>3. መንግስታዊ ያልሆነ ስራ/በግል የተቀጠረ<br>4. ነጋዴ<br>5. ተማሪ<br>6. የቤት እመቤት<br>7. የቤት ሰራተኛ<br>8. ጡረተኛ<br>9. ሌላ ካለ ይግለጹ________________	
106	ሀይማኖት	1. ኦርቶዶክስ<br>2. ሙስሊም<br>3. ፕሮቴስታንት<br>4. ካቶሊክ<br>5. ሌላ ካለ ይግለጹ _______________	
107	ብሔርዎ ምንድን ነዉ?	1. ሸካ<br>2. ከፋ<br>3. ቤንች<br>4. አማራ<br>5. ትግራይ<br>6. ኦሮሞ<br>7. ሌላ ካለ ይግለጹ ________________	
108	አጠቃላይ የቤተሰብ ወራዊ ገቢ ስንት ነዉ	______________________ ብር	

109	ለመጠጥ የሚጠቀሙትን ዉሃ የት ነዉ የሚያገኙት	1. ከወንዝ<br>2. ከምንጭ<br>3. ከጉድጓድ<br>4. ከቧንቧ<br>5. ሌላ ይግለጹ_______________________	
110	በቀን ምን ያክል ሊትር ዉሃ ይጠጣሉ	______________________ ሊትር	

ክፍል 2: ከሲጋራ እና አልኮል ጋር የተያያዙ ጥያቄዎች

111	በህይወት ዘመነዎ ሲጋ አጭሰዉ ያዉቃሉ	1. አወ<br>2. የለም	2 ከሆነ ወደ 114
112	ለስንት አመት አጭሰዋል	______________________አመት<br><br>2. አላሰታዉስም	
113	በቀን ምን ያክል ሲጋራ ያጨሳሉ	______________________የሲጋራ ብዛት	
114	በህይወት ዘመነዎ አልኮል ጠጥተዉ ያዉቃሉ	1. አወ<br>2. የለም	2 ከሆነ ወደ 117
115	ለስንት አመት ጠጥተዋል	______________________አመት<br><br>2. አላሰታዉስም	
116	በ ቀን ስንት ሊትር አልኮል ይጠጣሉ	______________________ሊትር	
117	ምግብ ላይ ጨው ይጨምራሉ	1. አልጨምርም<br><br>2. አልፎ አልፎ<br><br>3. አብዛኛውን ጊዜ<br><br>4. በእያንዳነዱ ምግብ	
118	የታሸጉ ምግቦችን ይመገባሉ	1. አልመገብም<br><br>2. አልፎ አልፎ<br><br>3. በሳምንት ትንሽ ጊዜ<br><br>4. በየቀኑ	

ክፍል 3: የአካል እንቅስቃሴ በተመለከተ

119	የምትሰራው ስራ ከበድ ያሉ እንቅስቃሴዎችን ቢያንስ ለ10 ደቂቃ የልብ እንቅስቃሴን ና ትንፋሽን የሚጨምር ስራን ያካትታል? (ለምሳሌ ከበድ ያሉ እቃዎችን ማንሳት ወይም መሸከም፤ መቆፈር፤ የግንባታ ስራ)	1. አዎ<br>2. አይደለም	2 ከሆነ ወደ 122

120	በሳምንት ውስጥ ለምን ያህል ቀን ከበድ ያሉ ስራዎችን ይሰራሉ?	የቀን ብዛት_______________	
121	ከበድ ያሉ ስራዎችን በመስራት በቀን ምን ያህል ሰዓት ያሳልፋሉ?	የሰዓታት<br>ብዛት______________<br>ደቂቃ______________	
122	የሚሰሩት ስራ መጠነኛ እንቅስቃሴዎችን ቢያንስ ለ10 ደቂቃ በመጠኑ የልብ እንቅስቃሴን ና ትንፋሽን የሚጨምር ስራን ያካትታል? (ለምሳሌ ቀለል ያሉ እቃዎችን ማንሳት)	1. አይደለም<br>2. አዎ	2 ከሆነ ወደ 125
123	በሳምንት ውስጥ ለምን ያህል ቀን የመጠነኛ እንቅስቃሴ ስራዎችን ይሰራሉ?	የቀን ብዛት______________	
124	የመለስተኛ እንቅሰቃሴ ስራዎችን በመስራት በቀን ምን ያህል ሰዓት ያሳልፋሉ?	የሰዓታት<br>ብዛት______________<br>ደቂቃ______________	

ከፍል 4: <u>የመዝናኛ እንቅስቃሴዎችን በተመለከተ</u>

125	ከበድ ያሉ እስፖርታዊ፤ የአካል ብቃት ወይም የመዝናኛ (የትርፍ ሰዓት) ዕንቅስቃሴዎች የልብ ዕናቅስቃሴንና ትንፋሽን በእጅጉ የሚጨምሩ እስፖርቶችን ቢያንስ ለ10 ደቂቃ በተከታታይ ትሰራለህ/ትሰሪያለሽ ?	1. አዎ<br>2. አይደለም	2 ከሆነ ወደ 128
126	በሳምንት ምን ያህል ቀናት ከበድ ያሉ እስፖርታዊ፤ የአካል ብቃት ና የመዝናኛ እንቅስቃሴዎችን ያደርጋሉ?	የቀን ብዛት______	
127	በቀን ከበድ ያሉ እስፖርታዊ፤ የአካል ብቃትና የመዝናኛ እንቅስቃሴዎችን በመስራት ለምን ያህል ሰዓት ይቆያሉ?	የሰዓታት ብዛት_____<br>ደቂቃ______	
128	መለስተኛ የሆኑ እስፖርታዊ፤ የአካል ብቃት ወይም የመዝናኛ (የትርፍሰዓት) ዕንቅስቃሴዎችን ወይም መለስተኛ የልብዕና ቅስቃሴን ና አተነፋፈስን የሚጨምሩ እስፖርቶችን ቢያንስ ለ10 ደቂቃ በተከታታይ ትሰራለህ/ትሰሪያለሽ?	1. አዎ<br>2. አይደለም	2 ከሆነ ወደ 131
129	በሳምንት ምን ያህል ቀናት መለስተኛ እስፖርታዊ፤ የአካል ብቃትና የመዝናኛ እንቅስቃሴዎችን ታደርጋለህ?	የቀን ብዛት______	
130	በቀን መለስተ እስፖርታዊ፤ የአካል ብቃትና የመዝናኛ እንቅስቃሴዎችን በመስራት ለምን ያህል ሰዓት ይቆያሉ?	የሰዓታት ብዛት_____<br>ደቂቃ______	

ከፍል 5: <u>የደም ግፊት ታሪከን በተመለከተ</u>

131	በደም ግፊት ታመዉ ያዉቃሉ ወይም የደም ግፊት መድሃት ወስደው ያውቃሉ	1. አዎ<br>2. አይደለም	2 ከሆነ ወደ 133
132	ለመጀመሪያ ጊዜ እንዳለብዎት ያወቁት መቼ ነዉ	1. ከአንድ አመት ወዲህ<br>2. ከ 1 እስከ 3 አመት[1-3)<br>3. ከ 3 እስከ 5 አመት[3-5)<br>4. ከ 5 እስከ 10 አመት[5-10)<br>5. ከ 10 በላይ(≥)	

133	ቤት ውስጥ የደም ግፊትዎትን ይለካሉ	1. አዎ<br>2. አይደለም	2 ከሆነ ወደ 136
134	ከለኩ ስንቴ	1. በየቀኑ<br>2. በሳምት አንዴ<br>3. በወርአንዴ<br>4. ሌላ(ይገለፕ)-----	
135	ለምን ያክል ጊዜ የደም ግፊት መጠን ከ140/90 በላይ ይሆናል	1.አብዛኛውን ጊዜ<br>2. አልፎ አልፎ<br>3. በጭራሽ ሁኖ አያዉቅም	
136	የአካል ብቃት እንቅስቃሴ ታደርጋለህ/ሽ(የደም ግፊት ለመቀነስ)	1. አዎ<br>2. አላደርግም	2 ከሆነ ወደ 138
137	ለጥያቄ ቁጥር 136 መልሱ አዎ ከሆነ፣ ለምን ያክል ጊዜ	1. በየቀኑ<br>2. በሳምንት 3 ቀን<br>3. በሳምንት 1 ቀን<br>4. በወር 1 ቀን<br>5. ሌላ(ይገለፕ)-----	
138	በድንገተኛ ሁኔታ ራስዎን ስተው (stroke) ያውቃሉ	1. አዎ<br>2. አይደለም	
139	የልብ በሽታ አሞዎት ያውቃል	1. አዎ<br>2. አይደለም	
140	ድንገተኛ የልብ ህመም አጋጥሞዎት ያውቃል	1. አዎ<br>2. አይደለም	
141	የደም ቧንቧ ቀዶ ጥገና አድርገው ያውቃሉ	1. አዎ<br>2. አይደለም	

ክፍል 6: <u>የኩላሊት በሽታን በተመለከተ</u>

142	የኩላሊት በሽታ አለብዎት ተብለው ያውቃሉ?	1. አዎ<br>2. አይደለም	2 ከሆነ ወደ 147
143	ለመጀመሪያ ጊዜ እንዳለብዎት ያወቁት መቼ ነዉ?	6. ከአንድ አመት ወዲህ<br>7. ከ 1 እስከ 3 አመት[1-3)<br>8. ከ 3 እስከ 5 አመት[3-5)<br>9. ከ 5 እስከ 10 አመት[5-10)<br>10. ከ 10 በላይ(≥)	
144	የኩላሊቱን በሽታ በምን ምክኒያት እንደመጣ ያውቃሉ	1. አዎ<br>2.. አላውቅም	
145	ምክኒያቱ ምን ነበር?	1. የስኳር በሽታ<br>2. ከፍተኛ የደም ግፊት<br>3. የኩላሊት ኢንፌክሽን<br>4. የኩላሊት ጠጠር<br>5. መድሃኒት<br>6. ቀዶ ጥገና<br>7. ሌላ (ይገለጥ)----------------	
146	ከሚከተሉት ውስጥ የነበሩብህ/ሽ ነበሩ<br><br>(ከአንድ በላይ መልስ ይቻላል)	1. በውልደት ወይም በልጂነት የመጣ የኩላሊት ችግር<br>2. በኩላሊት ስራ ማቆም ምክኒያት ሆሰፒታል መግባት<br>3. በሌላ ምክኒያት ሆስፒታል ገብቸ የኩላሊት በሽታ አለበወት ተብያለዉ<br>4. የኩላሊት ጠጠር<br>5. የሽንት ከረጢት ወይም የኩላሊት ኢንፌክሽን<br>6. ለመሽናት መቸገር<br>7. የሽንት ከረጢት ወይም ሌሎች የሽንት ቱቦ ቀዶ ጥገና<br>8. ወደ ሆድ እቃ ወይም ወደ ዳሌ ጨረር መለቀቅ<br>9. የካንሰር መድሃኒት<br>10. የቤተሰብ የኩላሊት በሽታ<br>11. ደም የቀላቀለ ሽንት	

ክፍል 7: <u>መድሃኒቶችን በተመለከተ</u>

147	ዘወትር ማሰታገሻ መድሃኒት ይወስዳሉ or NSAIDS (i.e. Aleve, naproxen, ibuprofen, Motrin)	1. አዎ<br>2. አልዎስድም	2 ከሆነ ወደ 149
148	ለጥያቄ ቁጥር 147 መልሱ አዎ ከሆነ በምን ያክል ጊዜ	1. በየቀኑ<br><br>2. በሳምንት 3 ቀን<br><br>3. በሳምንት 1 ቀን<br><br>4. በወር 1 ቀን<br><br>5. ሌላ(የገለፅ) _______	

149	የባህል መድሃኒት/ቅጠላቅጠል ይጠቀማሉ?	1. አዎ<br>2. አልዎስድም_______	2 ከሆነ ወደ 151
150	ለጥያቄ ቁጥር 149 መልሱ አዎ ከሆነ: ዘርዝር	ዝርዝሩን ከዚህ ያስቀምጡ	

ክፍል 8: የስኳር በሽታ ታሪክ በተመለከተ

151	የስኳር በሽታ አለብዎት ተብለው ያውቃሉ?	1. አዎ<br>2. አይደለም	2 ከሆነ 160
152	ለጥያቄ ቁጥር 151 መልሱ አዎ ከሆነ ለመጀመሪያ ጊዜ እንዳለብዎት ያወቁት መቼ ነዉ?	1. ከ 1 አመት ወዲህ<br>2. ከ 1 እስከ 5 አመት[1-5)<br>3. ከ 5 እስከ 10 አመት[5-10)<br>4. ከ 10 አመት በላይ(≥10)	
153	መድሀኒት ወይም ኢንሱሊን ወስደዋል	1. አዎ<br>2. አይደለም	
154	ለጥያቄ ቁጥር 153 መልሱ አዎ ከሆነ ለምን ያክል ጊዜ ወስደዋል	1. ከ 1 አመት ወዲህ<br>2. ከ 1 እስከ 5 አመት[1-5)<br>3. ከ 5 እስከ 10 አመት[5-10)<br>4. ከ 10 አመት በላይ(≥10)	
155	በአሁኑ ሰዓት መድሀኒት እየወሰዱ ነው	1. አዎ<br>2. አይደለም	
156	ለጥያቄ ቁጥር 155 መልሱ አይደለም ከሆነ ከመች ጀምሮ ነው ያቋረጡት	1. ከ 1 አመት ወዲህ<br>2. ከ 1 እስከ 5 አመት[1-5)<br>3. ከ 5 እስከ 10 አመት[5-10)<br>4. ከ 10 አመት በላይ(≥10)	
157	በቅርቡ የደም የስኳር መጠነወ ምን ያክል ነው	1. ከ 100 በታች(‹100)<br>2. ከ 100 እስከ 150[100-150]<br>3. ከ 150 እስከ 200(150-200]<br>4. ከ 200 በላይ(›200)	

		5. አላወቅኩም	
158	በስኳር በሽታ ምክኒያት የአይን በሽታ አጋጥሞት ያውቃል	1. አዎ<br><br>2. አይደለም	
159	በስኳር በሽታ ምክኒያት የእግር መደንዘዝ አጋጥሞት ያውቃል	1. አዎ<br><br>2. አይደለም	

ክፍል 9 : የደም ማነስ በሽታን በተመለከተ

160	የደም ማነስ በሽታ አለብዎት ተብለው ያውቃሉ?	1. አዎ<br><br>2. አይደለም	2 ከሆነ ወደ 164
161	ለጥያቄ ቁጥር 160 መልሱ አዎ ከሆነ ለመጀመሪያ ጊዜ እንዳለብዎት ያወቁት መቼ ነዉ?	1. ከአንድ አመት ወዲህ<br>2. ከ 1 እስከ 3 አመት[1-3)<br>3. ከ 3 እስከ 5 አመት[3-5)<br>4. ከ 5 እስከ 10 አመት[5-10)<br>5. ከ 10 በላይ(≥)	
162	የደም ማነስ በሽታን ለማዳን ወይም ለመከላከል መድሀኒት ወስደው ያውቃሉ	1. አዎ<br><br>2. አይደለም	
163	ለጥያቄ ቁጥር 162 መልሱ አዎ ከሆነ ምን አይነት<br><br>እባክዎ የተለያዩ ማሸጊያዎችን በማሳየት ያረጋግጡ<br><br>የወሰዱትን አይነት ያክብቡና የወሰዱትን መጠን ይጠይቁ	1. ፎሊክአሲድ፣ መጠን ________<br><br>2. አይረን፣ መጠን ________<br><br>3. ቫይተሚን B12 ________<br><br>4. Epogen or Aranesp_______<br><br>5. ሌላ(ይጠቀስ)________	
164	የአይነ ምድር መጥቆር አጋጥሞት ያውቃል	1. አዎ<br><br>2. አይደለም	
165	በአይነ ምድር ውስጥ ደም ተመልክተው ያውቃሉ	1. አዎ<br><br>2. አይደለም	
166	በሽንት ውስጥ ደም ተመልክተው ያውቃሉ	1. አዎ<br><br>2. አይደለም	
167	በቤተሰብ ውስጥ/የቅረብ ዘምድ የደም ማነስ በሽታ ያለበት ሰው አለ	1. አዎ	

		2. አይደለም	
168	ሴት ከሆኑ፣ እስካሁን የወር አበባ ታያለሽ	1. አዎ<br><br>2. አይደለም	2 ከሆነ ወደ 170
169	ለጥያቄ ቁጥር 168 መልሱ አዎ ከሆነ በየስንት ጊዜ/ወር	_______ ወር	
170	ከሚከተሉት ውስጥ የትኛውን በሽታ እንዳለብዎት ተነግሮት ያውቃል	1. Lymphoma<br>2. የደም ካንሰር<br>3. ደም ማስመለስ<br>4. የሆድ ቁስለት<br>5. ተደጋጋሚ የሆነ ነስር<br>6. ሌሎች ካንሰር______________	

ክፍል 10፡ የሰውነት ልከት በተመለከተ

171	ቁመት	በ ሳ.ሜ________________
172	ክብደት	በ ኪ.ግ________________
173	የወገብ ዙሪያ መጠን	በ ሳ.ሜ_____________
174	የደም ግፊት መጠን	በ ሳ.ሜ________________
	ንባብ 1	Systolic(mmHg) _____________
		Diastolic (mmHg) ___________
	ንባብ 2	Systolic(mmHg) _____________
		Diastolic (mmHg) ___________

ክፍል 11 ፡ የላቦራቶሪ ልኬታን በተመለከተ

175	Proteinuria	1. ፖዘቲቭ<br>2. ኔጋቲቭ
176	Hematuria	1. ፖዘቲቭ<br>2. ኔጋቲቭ
177	የደም ስኳር መጠን	_____________mg/dl<br>_____________mmol/L
178	Creatinine level	_____________mg/dl or<br>_____________mmol/L
179	GFR	_____________ml/min/1.73m^2

አመሰግናለሁ!
