# Supplementary material for: Chronic kidney disease and associated factors among adult population in Southwest Ethiopia
Source: PLoS One. 2022 Mar 3;17(3):e0264611. doi: 10.1371/journal.pone.0264611 (PMC8893675; doi:10.1371/journal.pone.0264611)
Supplement: Attachment — Submitted filename: PONE-D-20-08526_R1_reviewer (1).pdf [file pone.0264611.s004.pdf]

PLOS ONE

Chronic Kidney Disease and Associated Factors among Adult Population in Southwest Ethiopia

--Manuscript Draft--

**Manuscript Number:**	PONE-D-20-08526R1
**Article Type:**	Research Article
**Full Title:**	Chronic Kidney Disease and Associated Factors among Adult Population in Southwest Ethiopia
**Short Title:**	Chronic Kidney Disease and Associated Factors among Adult Population in Southwest Ethiopia
**Corresponding Author:**	Dejene Derseh, MSc<br>Mizan Tepi University<br>Mizan Tefri, SNNPR ETHIOPIA
**Keywords:**	Chronic kidney disease, Associated factors, Adult, Ethiopia
**Abstract:**	In Ethiopia, data on the burden and ~~determinates~~ of chronic kidney disease (CKD) in the general population is limited. This community-based study was conducted to assess the burden and associated factors of CKD among adults in Southwest Ethiopia. The study was conducted from August 23, 2018-October 16, 2018. Study participants were selected using a random sampling method. A structured questionnaire was used to collect relevant data. Blood pressure and anthropometric ~~measurements~~ were ~~made~~ following standard procedures. About 5 ml of urine sample was collected and the dipstick test was performed immediately. A blood sample of 3-5ml was collected for serum creatinine and blood glucose level determination. The three commonest estimators of glomerular filtration rate and the National Kidney Foundation Kidney Disease Outcomes Quality Initiative were used to define and stage CKD. Data were analyzed using SPSS 21. Multivariable logistic regression was employed and p-value <0.05 was used to indicate statistically significant results. A total of 326 participants with a mean age of 39.9(SD±11.2) years were enrolled in the study. The proportions of female participants (59.8%) were relatively higher than male participants (40.2%). The mean eGFR using CKD-EPI, CG and MDRD was 124.34 (SD±23.8), 110.67(SD±33.0) and 131.29 (SD±32.5) respectively. The prevalence of CKD was 7.4% using CKD-EPI ~~&~~ MDRD and 8% using CG. Similar finding using CKD-EPI ~~&~~ MDRD may indicate that either CKD-EPI or MDRD can be used to estimate GFR in this study area. In the age and sex-adjusted logistic regression model, hypertension was significantly associated with CKD using CKD-EPI ~~&~~ MDRD and age ≥40 years old was significantly associated with CKD using CG. Behavioral characteristics and other traditional risk factors were not significantly associated with CKD in the current study. The prevalence of CKD was comparable with other sub-Saharan African countries. Only hypertension and age ≥40 years old were significantly associated with CKD. More of the increased prevalence of CKD in the current study remained unexplained and deserves further study.
**Order of Authors:**	Kindie Mitiku Kebede
	Dejene Derseh Abateneh, MSc
	Melkamu Beyene Teferi
	Abyot Asres
**Response to Reviewers:**	Ref No: PONE-D-20-08526<br>Title: Chronic Kidney Disease and Associated Factors among Adult Population in Southwest Ethiopia<br>Response to reviewers<br>1.We recommend to include the potential impact of the kebeles in the results of the survey.<br>Response: Dear reviewer, all the kebeles included in this study were urban kebeles and assumed to be homogenous. Therefore, the prevalence of CKD is not expected to vary among the included kebeles.<br>2.We require to suppress these first lines and start the paper directly with the CKD.<br>Response: We have deleted the first paragraph and started the paper directly with the

Powered by Editorial Manager® and ProduXion Manager® from Aries Systems Corporation

CKD (Introduction section, page 2, line 38-44).
3.Authors are required to re-define what advanced age means or modify the age ranges. > 40 years cannot be considered advanced age
Response: Dear reviewer thank you very much for this comment. We couldn't find a standard definition for advanced age for sub-Saharan countries. For this reason, we have removed the term "advanced age" and simply we categorized age as ≥40 years old and <40 years old. The age category was decided for comparison (Compare with other studies) (Abstract section, page 2, line 30; Discussion section, page 25, line 316; Conclusions section, page 26, line 337and 341).
4.The increased prevalence of CKD in the current study remains unexplained and scarcely discussed. Authors are required to recommend the use of CKD-EPI, MDRD or CG to estimate CKD prevalence in order to make recommendations.
Response: Even though the aim of this study is not to recommend the use of CKD-EPI, MDRD or CG, we have incorporated recommendations on the use of CKD-EPI, MDRD, or CG to estimate CKD prevalence in this study area (Abstract section, page 2, line 27-28).
5.The paper is suitable for publication but needs to focus in the novelty and originality of the studied population.
Response: Dear reviewer, thank you for this comment. At this stage, we can't change the study population. It is difficult to say that the study population is unique. This study was conducted among the urban population in the Sheka zone. Therefore, the population may share similar characteristics with other urban populations in Ethiopia. However, this study is the first community-based study in the study area and in Ethiopia at large. In fact, this study has certain limitations. We have included those limitations in the manuscript.
Response to Editor
1.There are similar papers as Sumaili et al in 2009 about prevalence of Chronic Kidney Disease in the Democratic Republic of Congo. Although there is no significant new clinical data, the study is based in a survey and there are some socio-demographic data that are of interest. The survey is based in an administrative Ethiopian distribution of kebeles, making it original. However, these points are not discussed along the paper.
Response: Dear editor, all the kebeles included in this study were urban kebeles and assumed to be homogenous. Therefore, the prevalence of CKD is not expected to vary among the included kebeles.
2.It would be of interest to compare the urban and rural areas.
Response: Dear editor, this study was exclusively studied in urban areas. Therefore, it is difficult to compare urban and rural areas.
3.Moreover, the introduction is initially focused in the global burden of noncommunicable diseases (NCD) but this not the main point along the paper.
Response: We have deleted the first paragraph and started the paper directly with the CKD (page 2, line 38-44).
4.Hypertension and advanced age were significantly associated with the excess risk of CKD, however the advanced age criteria is > 40 years. Authors must re-define what advanced age means or modify the age ranges.
Response: Dear editor, thank you very much for this comment. We couldn't find a standard definition for advanced age for sub-Saharan countries. For this reason, we removed the term "advanced age" and simply we categorized age as ≥ 40 years old and <40 years old. The age category was decided for comparison (Compare with other studies) (Abstract section, page 2, line 30; Discussion section, page 25, line 316; Conclusions section, page 26, line 337 and 341).
5.The increased prevalence of CKD in the current study remains unexplained and scarcely discussed. Authors must recommend the use of CKD-EPI, MDRD or CG to estimate CKD prevalence in order to make recommendations.
Response: Even though the aim of this study is not to recommend the use of CKD-EPI, MDRD, or CG, we have incorporated recommendation on use of CKD-EPI, MDRD or CG to estimate CKD prevalence in this study area (page 2, line 27-28)
6.The paper is suitable for publication but needs to focus in the novelty and originality of the studied population.
Response: Dear editor, thank you for this comment. At this stage, we can't change the study population. It is difficult to say that the study population is unique. This study was conducted among the urban population in the Sheka zone. Therefore, the population may share similar characteristics with other urban populations in Ethiopia. However, this study is the first community-based study in the study area and in Ethiopia at large.

Powered by Editorial Manager® and ProduXion Manager® from Aries Systems Corporation

	In fact, this study has certain limitations. We have included those limitations in the manuscript.<br>7.I consider that after this correction in the introduction (eliminate the initial lines related to NCD, and start with CKD prevalence and the collected data related with general and CKD populations<br>Response: Corrected based on your comment.<br>8.If you developed a questionnaire as part of this study and it is not under a copyright more restrictive than CC-BY, please include a copy, in both the original language and English, as Supporting Information.<br>Response: We have uploaded a questionnaire both in English and local language (Amharic) as supporting information.
**Additional Information:**	
**Question**	**Response**
**Financial Disclosure**<br><br>Enter a financial disclosure statement that describes the sources of funding for the work included in this submission. Review the submission guidelines for detailed requirements. View published research articles from *PLOS ONE* for specific examples.<br><br>This statement is required for submission and **will appear in the published article** if the submission is accepted. Please make sure it is accurate.<br><br>**Unfunded studies**<br>Enter: *The author(s) received no specific funding for this work.*<br><br>**Funded studies**<br>Enter a statement with the following details:<br>• Initials of the authors who received each award<br>• Grant numbers awarded to each author<br>• The full name of each funder<br>• URL of each funder website<br>• Did the sponsors or funders play any role in the study design, data collection and analysis, decision to publish, or preparation of the manuscript?<br>• **NO** - Include this sentence at the end of your statement: *The funders had no role in study design, data collection and analysis, decision to publish, or preparation of the manuscript.*<br>• **YES** - Specify the role(s) played.<br><br>* typeset	Funding for this study was made possible through grants offered by Mizan Tepi University, Ethiopia with grant number MTURD/3268/18). The award was received by the author Mr. Kindie Mitiku Kebede. The funder had no role in study design, data collection and analysis, decision to publish, or preparation of the manuscript.
**Competing Interests**	The authors have declared that no competing interests exist.

Use the instructions below to enter a competing interest statement for this submission. On behalf of all authors, disclose any competing interests that could be perceived to bias this work—acknowledging all financial support and any other relevant financial or non-financial competing interests.<br><br>This statement **will appear in the published article** if the submission is accepted. Please make sure it is accurate. View published research articles from *PLOS ONE* for specific examples.<br><br>**NO authors have competing interests**<br><br>Enter: *The authors have declared that no competing interests exist.*<br><br>**Authors with competing interests**<br><br>Enter competing interest details beginning with this statement:<br><br>*I have read the journal's policy and the authors of this manuscript have the following competing interests: [insert competing interests here]*<br><br>* typeset	
**Ethics Statement**<br><br>Enter an ethics statement for this submission. This statement is required if the study involved:<br><br>• Human participants<br>• Human specimens or tissue<br>• Vertebrate animals or cephalopods<br>• Vertebrate embryos or tissues<br>• Field research<br><br>Write "N/A" if the submission does not require an ethics statement.<br><br>General guidance is provided below. Consult the submission guidelines for detailed instructions. **Make sure that all**	Ethical approval was obtained from the ethics committee of Mizan Tepi University research directorate (Ref No: MTU/27/514/44/10). Permission of Sheka Zone, Southwest Ethiopia Health office and the head of the woreda were guaranteed. After explaining the objectives of the study, written consent was obtained from each study participant. For the participants who were not able to read and write, a fingerprint was used as a signature. The referral was considered for study participants with CKD and other medical risk factors.

Powered by Editorial Manager® and ProduXion Manager® from Aries Systems Corporation

**information entered here is included in the Methods section of the manuscript.**<br><br>**Format for specific study types**<br><br>**Human Subject Research (involving human participants and/or tissue)**<br>• Give the name of the institutional review board or ethics committee that approved the study<br>• Include the approval number and/or a statement indicating approval of this research<br>• Indicate the form of consent obtained (written/oral) or the reason that consent was not obtained (e.g. the data were analyzed anonymously)<br><br>**Animal Research (involving vertebrate animals, embryos or tissues)**<br>• Provide the name of the Institutional Animal Care and Use Committee (IACUC) or other relevant ethics board that reviewed the study protocol, and indicate whether they approved this research or granted a formal waiver of ethical approval<br>• Include an approval number if one was obtained<br>• If the study involved *non-human primates*, add *additional details* about animal welfare and steps taken to ameliorate suffering<br>• If anesthesia, euthanasia, or any kind of animal sacrifice is part of the study, include briefly which substances and/or methods were applied<br><br>**Field Research**<br><br>Include the following details if this study involves the collection of plant, animal, or other materials from a natural setting:<br>• Field permit number<br>• Name of the institution or relevant body that granted permission	
**Data Availability**<br><br>Authors are required to make all data underlying the findings described fully available, without restriction, and from the time of publication. PLOS allows rare exceptions to address legal and ethical	Yes - all data are fully available without restriction

concerns. See the PLOS Data Policy and FAQ for detailed information.<br><br>A Data Availability Statement describing where the data can be found is required at submission. Your answers to this question constitute the Data Availability Statement and **will be published in the article**, if accepted.<br><br>**Important:** Stating 'data available on request from the author' is not sufficient. If your data are only available upon request, select 'No' for the first question and explain your exceptional situation in the text box.<br><br>Do the authors confirm that all data underlying the findings described in their manuscript are fully available without restriction?	
**Describe where the data may be found in full sentences. If you are copying our sample text, replace any instances of XXX with the appropriate details.**<br><br>• If the data are **held or will be held in a public repository**, include URLs, accession numbers or DOIs. If this information will only be available after acceptance, indicate this by ticking the box below. For example: *All XXX files are available from the XXX database (accession number(s) XXX, XXX.*).<br>• If the data are all contained **within the manuscript and/or Supporting Information files**, enter the following: *All relevant data are within the manuscript and its Supporting Information files.*<br>• If neither of these applies but you are able to provide **details of access elsewhere**, with or without limitations, please do so. For example:<br><br>*Data cannot be shared publicly because of [XXX]. Data are available from the XXX Institutional Data Access / Ethics Committee (contact via XXX) for researchers who meet the criteria for access to confidential data.*	All relevant data are within the manuscript and its Supporting Information files.

Powered by Editorial Manager® and ProduXion Manager® from Aries Systems Corporation

The data underlying the results presented in the study are available from (include the name of the third party and contact information or URL).<br>• This text is appropriate if the data are owned by a third party and authors do not have permission to share the data.<br><br>* typeset	
Additional data availability information:	

Powered by Editorial Manager® and ProduXion Manager® from Aries Systems Corporation

Manuscript Click here to access/download;Manuscript;Manuscript.docx

Chronic Kidney Disease and Associated Factors among Adult Population in Southwest Ethiopia

Kindie Mitiku Kebede[1], Dejene Derseh Abateneh[1,2]*, Melkamu BeyeneTeferi[1], Abyot Asres[1]

[1]College of Medicine and Health Sciences, Mizan Tepi University, Mizan Teferi, Ethiopia.

[2]Menelik II College of Medicine and Health Sciences, Kotebe Metropolitan University, Addis Ababa, Ethiopia.

***Corresponding author:**

Mailing address: Kotebe Metropolitan University, Menelik II College of Medicine and Health Sciences, Department of Medical Laboratory Sciences, Addis Ababa, Ethiopia. E-mail: dejenieh@gmail.com, P.O.BoX: 3268. Tel: +251920514158.

Abstract

In Ethiopia, data on the burden and ~~determinates~~ of chronic kidney disease (CKD) in the general population is limited. This community-based study was conducted to assess the burden and associated factors of CKD among adults in Southwest Ethiopia. The study was conducted from August 23, 2018-October 16, 2018. Study participants were selected using a random sampling method. A structured questionnaire was used to collect relevant data. Blood pressure and anthropometric ~~measurements~~ were ~~made~~ following standard procedures. About 5 ml of urine sample was collected and the dipstick test was performed immediately. A blood sample of 3-5ml was collected for serum creatinine and blood glucose level determination. The three commonest estimators of glomerular filtration rate and the National Kidney Foundation Kidney Disease Outcomes Quality Initiative were used to define and stage CKD. Data were analyzed using SPSS 21. Multivariable logistic regression was employed and p-value <0.05 was used to indicate statistically significant results. A total of 326 participants with a mean age of 39.9(SD±11.2) years were enrolled in the study. The proportions of female participants (59.8%) were relatively higher

than male participants (40.2%). The mean eGFR using CKD-EPI, CG and MDRD was 124.34 (SD±23.8), 110.67(SD±33.0) and 131.29 (SD±32.5) respectively. The prevalence of CKD was 7.4% using CKD-EPI & MDRD and 8% using CG. Similar finding using CKD-EPI & MDRD may indicate that either CKD-EPI or MDRD can be used to estimate GFR in this study area. In the age and sex-adjusted logistic regression model, hypertension was significantly associated with CKD using CKD-EPI & MDRD and age ≥40 years old was significantly associated with CKD using CG. Behavioral characteristics and other traditional risk factors were not significantly associated with CKD in the current study. The prevalence of CKD was comparable with other sub-Saharan African countries. Only hypertension and age ≥40 years old were significantly associated with CKD. More of the increased prevalence of CKD in the current study remained unexplained and deserves further study.

Keywords: Chronic kidney disease, Associated factors, Adult, Ethiopia

Introduction

Chronic kidney disease (CKD) is among Non-communicable diseases (NCD) which cause significant morbidity and mortality globally [1]. A recent systematic review and meta-analysis estimated that the global pooled prevalence of CKD ranges from 11–13% and 13.9% prevalence in sub-Saharan Africa [2]. The epidemiology of CKD in sub-Saharan Africa is different from other regions that it mainly affects the economically productive young age group. The risk of CKD like HIV infection, hypertension, diabetes, and other infectious diseases are increasing in sub-Saharan Africa [2-5].

Although Ethiopia is among the countries that strive to achieve sustainable development goals 2016-2030, the progress towards the reduction of NCD associated deaths is limited. More than one

third of, (39%) annual deaths are associated with NCD [6]. The burden of CKD is increasing and mainly associated with poor community awareness, insufficient data, and poor healthcare infrastructure [7]. In Ethiopia, studies that assess the prevalence and associated factors of CKD are very limited. The available studies are not representative because they are taken from high-risk groups, like, diabetes and HIV patients [7,8]. A high prevalence of CKD, 18.2% and 23.8% using the Modification of Diet in Renal Disease (MDRD) and Cockcroft-Gault (C-G) equations respectively was reported among diabetic patients [7]. Owing to limited community based published reports available so far on CKD in Ethiopia, this study was conducted to assess the prevalence and associated factors of CKD among adults in Southwest Ethiopia.

Materials and Methods

Study area, study design, and participants

A community-based cross-sectional study was conducted in the Sheka zone, Southern Nation, Nationality and People Region (SNNPR), Southwest Ethiopia. It is bordered on the South by Bench Maji, on the West by the Gambella region, on the North by the Oromia region and on the East by the Kaffa zone. The administrative center of Sheka is Masha which is located approximately 610 Km Southwest of Addis Ababa. Based on the 2007 census conducted by the central statistical agency, the zone had a total population of 199,314 of whom 101,059 were men and 98,255 were women. About 34, 2227 (17.17%) were urban inhabitants.

The study was conducted from August 23, 2018 to October 16, 2018. The minimum sample size (N) was determined by using single population proportion formula [$N = (Z\ \alpha/2)^{2} P (1-P) /d^{2}$], where $Z_{\alpha/2}$ = the value under standard normal table at 95% level of confidence which is 1.96, prevalence P, 15.2% prevalence of CKD among urban adults [9], margin of error d, which was set at 4%. Including a 10% non-response rate, the final sample size was 384 adults. Permanent

residents aged ≥18 years in the households of Masha and Tepi town were included in the study. However, pregnant women and participants with acute illness with fever during data collection were excluded. Mentally disabled and those unable to give response were also excluded from the study.

Sampling procedure

A total of two urban areas of Sheka zone was selected randomly i.e. Tepi and Masha from a total of three urban areas (Tepi, Mash and Andracha). Tepi town had three kebeles, while Masha town had two kebeles during the study period. A total of 3 kebeles were randomly included in the study from a total of 5 kebeles in the selected urban areas. Then, the lists of households were taken from the administration of each kebele. All households in the selected urban kebeles with adults ≥18 years old were enumerated with the help of health extension workers to generate the sampling frame. After the generation of the sample framing, the sample size was allocated proportionally to the total household of each kebele. Finally, eligible participants were selected using a random sampling method. In the case of more than one adult population in a single household, one was selected using the lottery method. In case where eligible respondents were not available at the time of data collection, a revisit for three times was made. Respondents who were not available after three visits were considered as non-respondents.

Data collection and laboratory methods

A structured questionnaire adapted from various literatures [9-11] was used to collect data on socio-demographic, economic, behavioral and co-morbidity related characteristics of participants. Height, weight, blood pressure and waist circumference of all participants were measured by trained data collectors following standardized procedures. Blood pressure readings were performed using a digital blood pressure apparatus after the participant had remained in a sited

position for three to five minutes on his or her right arm resting on the chair. Weight, height and waist circumference were measured in light clothing without shoe. Height was taken to the nearest 0.1 cm with a portable stadiometer (Seca 274, Germany) and weight was measured to the nearest 0.1k.g on mechanical Seca (Seca761, Germany). Waist circumference was measured using tape measures on the midway between the top of the hip bone and the bottom of the ribs. Participants were instructed to put off their heavy clothes and breathe out normally before waist circumference measurements. Waist circumference measurements were taken to the nearest 0.1cm. Blood pressure and all anthropometric measurements were made twice, and their averages were used in all analysis.

About 5 ml random urine sample was collected using a clean, leak-proof urine cup for urine chemical analysis. Chemical analysis of urine specimens was performed immediately after sample collection using urine dipsticks test (Multistix® Henry Schein, Inc. https://www.henryschein.com/medical-multistix.aspx). Semi-quantitative chemical analysis of CKD markers (protein) was performed.

Blood sample of 3 - 5 ml for serum creatinine and blood glucose level determination was collected using a syringe with needle and transferred to a gel containing serum separator test tube. Blood specimens were transported to the nearest general hospital (Tepi general hospital) for serum separation. A serum was separated after the sample clotted and centrifuged at 1000 – 2000g for 10 minutes by trained laboratory technologist. Serum sample was immediately separated from the whole blood and transferred to nunc tube. The serum was kept frozen at -20 ºC until processed and then transported on ice-cooled containers to Jimma teaching and referral hospital, Ethiopia for analysis.

The creatinine and blood glucose levels were measured using the ABX pentra400 chemistry analyzer and reported in mg/dL or µmol/L (HORIBA, ABX, Japan). The ABX Pentra 400 is a multiparametric analyzer in which it has good reliability and practicability for routine and specialized clinical chemistry analyses, evaluated according to the National Committee for Clinical Laboratory Standards [12].

Variables' definition

The three common equations were used to estimate glomerular filtration rate (eGFR), and the National Kidney Foundation Kidney Disease Outcomes Quality Initiative (NKF/KDOQI) guidelines to define and stage CKD [13]. We defined a participant as having CKD if he or she had GFR below 60 ml/min/1.73 m^2 and/or had proteinuria (positive dipstick at least +1). Glomerular filtration rate classification was based on the following: stage 1: GFR > 90 with proteinuria; stage 2: GFR 60 – 89 with proteinuria; stage 3a: GFR 45 – 59; stage 3b: GFR 30 – 44; stage 4: GFR 15 – 29 and stage 5 end-stage renal disease (ESRD): GFR < 15 (Fig 1). Hypertension was defined as a systolic (SBP) ≥140 mmHg and/or a diastolic blood pressure of (DBP) ≥90 mmHg or self-reported on anti-hypertension drugs. Diabetes was defined as random blood sager >200mg/dl and/or self-reported on anti-hyperglycemic medication. A separate cut of points was used to level central obesity for male men and women: women were leveled as obese if their waist circumference was ≥88 cm and men were leveled as obese if their waist circumference ≥102 cm. The nutritional status of participants was assessed using body mass index (BMI) and leveled as follows: underweight: <18.5; normal weight: 18.5 – 24.9; overweight: 25 – 29.9 and obese: >30.

Traditional causes of CKD: Refer to the well-known causes of CKD such as hypertension, diabetes, obesity, and HIV.

Heart diseases: In this study, it means a self-reported history of heart diseases such as coronary disease (vessel surgery), or heart failure, or heart attack or stroke.

Moderate activities/exercise: Brisk walking or carrying light loads, gardening, household /domestic chores, roofing, painting for 30 minutes per day for five days a week

Vigorous exercise/activity: Carrying or lifting heavy loads, digging or construction work, running, fast cycling, aerobics, fast swimming, playing football & volleyball for 30 minutes per day for five days a week.

Fig 1. Definitions and stages of Chronic Kidney Disease [13].

Data analysis and interpretation

Data were entered into Epidata version 3.1 and exported to SPSS 21.0 for further analysis. Continuous data were presented as mean (± standard deviation) and categorical data as proportions. We used chi-square and fishers exact test for comparison of proportions and independent t-test for comparing mean differences. Logistic regression models were used to investigate the predictors of CKD. Variables with p-value of <0.2 in the bivariate analysis were included in the multivariable analysis. A p-values <0.05 were used to indicate statistically significant results.

Data quality control

Data collectors were trained and supervised during the data collection process. The questionnaire was first prepared in the English language and translated to the local language (Amharic). About 10% of the questionnaire was pretested among urban Kebeles of Sheka zone which were not included in this study before the actual study period. Completeness and consistency of the collected data were checked during data collection by the supervisors. Anthropometric equipment's were calibrated frequently for every five measurements to control fallacy. Both intra-observer and

interobserver variations were checked using SPSS version 21.0 during training of anthropometric measurement and the training was continued until an acceptable level of variation reached. Laboratory instruments were calibrated to guarantee reliability test result and laboratory tests were done according to manufacturer's instructions, with their respective controls.

Ethics approval and consent to participate

Ethical approval was obtained from the ethics committee of Mizan Tepi University research directorate (Ref No: MTU/27/514/44/10). Permission of Sheka Zone, Southwest Ethiopia Health office and the head of the woreda were guaranteed. After explaining the objectives of the study, written consent was obtained from each study participant. For the participants who were not able to read and write, a fingerprint was used as a signature. The referral was considered for study participants with CKD and other medical risk factors.

Results

Socio-demographic characteristics

A total of 326 participants which gives a 95.9% response rate were included in this study. Most participants were within the age category of 18-39 (241; 73.9%) years. The proportions of female participants (59.8%) were relatively higher than male participants (40.2%). Significant differences in age, marital status and occupation were observed between male and females (p<0.05) as indicated in Table 1.

Table 1. Socio-economic and demographic characteristics of study participants in Sheka zone, Southwest Ethiopia, 2018.

**Characteristics**	**Overall**	**Men**	**Women**	**p-value**
**Age (**Mean=33.90 ± 11.2 years)				0.011
18–39 years old	241(73.9)	87(66.4)	154(79.0)	
≥ 40 years old	85(26.1)	44(33.6)	41(21.0)	
**Education status**				0.163

Not attended formal education	53(16.3)	17(13.0)	36(18.5)	
Primary (1-8)	116(35.6)	42(32.1)	74(37.9)	
Secondary (9-12)	90(27.6)	44(33.6)	46(23.6)	
Above (12+)	67(20.6)	28(21.4)	39(20.0)	
**Marital status**				0.032
Married	267(81.9)	100(76.3)	167(85.6)	
Others*	59(18.1)	31(23.7)	28(14.4)	
**Religion**				0.477
Christian	242(74.2)	100(41.3)	31(36.9)	
Muslim	84(25.8)	142(58.7)	53(63.1)	
**Occupation**				<0.001
Employed**	53(16.3)	22(16.8)	31(15.9)	
Farming	56(17.2)	37(28.2)	19(9.7)	
Merchant	49(15.0)	36(27.5)	13(6.7)	
Housewife	104(31.9)	3(2.3)	101(51.8)	
Students	42(12.9)	18(13.7)	24(12.3)	
Others***	22(6.7)	15(11.5)	7(3.6)	
**Monthly income ((**Median 2000(IQR= 2200 EB))				0 .110
<2000EB	113(34.7)	38(36.9)	75(46.9)	
≥2000EB	150(46.0)	65(63.1)	85(53.1)	
**Main source of water for drinking**				0.771
Pip water	171(52.5)	70(53.4)	101(51.8)	
Wall/spring	155(47.5)	61(46.6)	94(48.2)	

EB =Ethiopian birr*Single, divorced or widowed ** Private and government employed ***house maid, retired, unemployed

Behavioral and Lifestyle characteristics

Majority of participants didn't engage in vigorous activity/exercise (83.1%) or moderate activity/exercise (72.4%). A very small proportion (3.1%) of participants ever used herbal medicines. About 43 (13.2%) of the participants reported that they used nonsteroidal anti-inflammatory drugs. Nearly one-third of the participants (34.0%) reported that they drink <2 liters of water per day. A significant difference in alcohol and cigarette consumption, moderate exercise/activity, vigorous exercise/activity, and consumption of canned food between males and female were observed as described in Table 2.

Table 2. Behavioral related characteristics of study participants in Sheka zone, Southwest Ethiopia, 2018.

Characteristics	Overall	Men	Women	P-value
**Ever drink alcohol**				<0.001
Yes	46(14.1)	34(26.0)	12(6.2)	
No	280(85.9)	97(74.0)	183(93.8)	
**Ever smoke cigarette**				
Yes	17(5.2)	17(13.0)	0(0.0)	
No	309(94.8)	114(87.0)	195(100.0)	
**Smoking year**				0.098
<10 years	13(76.5)	49(37.4)	91(46.7)	
≥10 years	4(23.5)	82(62.6)	104(53.3)	
**Herbal medicine intake**				0.326 #
Yes	10(3.1)	2(1.5%)	8(4.1)	
No	316(96.9)	129(98.5)	187(95.9)	
**Vigorous exercise/activity**				0.037
Yes	55(16.9)	29(22.1)	26(13.3)	
No	271(83.1)	102(77.9)	169(86.7)	
**Moderate exercise/activity**				0.013
Yes	90(27.6)	46(35.1)	44(22.6)	
No	236(72.4)	85(64.9)	151(77.4)	
**Added salt to food**				0.931
Never	18(5.5)	8(6.1)	10(5.1)	
Occasionally	55(16.9)	22(16.8)	33(16.9)	
With each meal	253(77.6)	101(77.1)	152(77.9)	
**Eat canned/processed food**				<0.001
Never	193(59.2)	63(48.1)	130(66.7)	
Yes (occasionally and often)	133(40.8)	68(51.9)	65(33.3)	
**Daily water intake**				
<2 letters per day	111(34.0)	46(35.1)	65(33.3)	0.739
≥ 2 letters per day	215(66.0)	85(64.9)	130(66.7)	
**Use of over NSAIDs drugs regularly**				0.002
Yes	43(13.2)	8(6.1)	35(17.9)	
No	283(86.8)	123(93.9)	160(82.1)	

Fishers exact test was used

Clinical characteristics/Co-morbidities

One fifth (21.1%; n=71) of the participants were hypertensive: defined as being on antihypertensive drug or having a systolic blood pressure of ≥140mmHg and /or diastolic blood pressure of ≥ 90mmHg. Similarly, one-fifth of the participants (20.2%; n=66) reported history of anemia. However, small proportion of the participants (3.4%; n=11) were diabetic defined as being on drugs for diabetes and/or having blood glucose level >200mg/dl. Overall, there is a significant difference in the proportion of self-reported history of anemia and kidney stone between men and females (p<0.05) as described in Table 3.

Table 3. Clinical/co-morbidity related characteristics of study participants in Sheka zone, Southwest Ethiopia, 2018.

Characteristics	Overall	Men	Women	P-value
**Hypertension**				0.215
Yes	71(21.8)	24(18.3)	47(24.1)	
No	255(78.2)	107(81.7)	148(75.9)	
**Diabetes**				0.761#
Yes	11(3.4)	5(3.8)	6(3.1)	
No	315(96.6)	126(96.2)	189(96.9)	
**Anemia**				<0.001
Yes	66(20.2)	6(4.6)	60(30.8)	
No	260(79.8)	125(95.4)	135(69.2)	
**Heart Disease***				0.374 #
Yes	12(3.7)	3(2.3)	9(4.6)	
No	314(96.3)	128(97.7)	186(95.4)	
**Kidney stone**				0.049
Yes	34(10.4)	19(14.5)	15(7.7)	
No	292(89.6)	112(85.5)	180(92.3)	

* Heart Disease includes coronary disease (vessel surgery), heart failure, or heart attack or stork, # Fishers exact test was used

Anthropometric and laboratory measurements

Table 4 describes the mean differences of the anthropometric measurements and laboratory tests between men and women which were assessed using an independent t-test. A significant mean difference in height, BMI, serum creatinine, and eGFR using CKD-EPI and CG were observed between men and women ($p<0.05$). As expected, serum creatinine was higher in men than women ($p < 0.001$) but they had a significantly lower mean estimated creatinine clearance. We also found significantly high level of obesity among women as compared to men ($p<0.001$).

Table 4. Anthropometric and laboratory measurement results of study participants in Sheka zone, Southwest Ethiopia, 2018.

**Characteristics**	**Overall**	**Men**	**Women**	**P-value**
**Mean weight (Kg.)**	60.96±9.63	61.49± 9.13	60.61±9.96	0.414
**Mean height(meter)**	1.60± 0.08	1.66±0.08	1.57±0.06	<0.001
**Mean SBP, mmHg (SD)**	121.01±15.1	121.14±12.5	120.92±16.6	0.896
**Mean DBP, mmHg (SD)**	80.11± 10.7	79.30±9.2	80.66±11.6	0.262
**Mean BMI, kg/m2 (SD)**	23.84±4.10	22.52±3.7	24.73±4.1	<0.001
**Mean serum creatinine, mg/dl (SD)**	0.74±0.22	0.99±.31	0.69±.14	<0.001
**Mean creatinine clearance, ml/min (SD)**				
MDRD	131.29±32.5	114.79±37.6	135.03±30.5	0.075
CG	110.67±33.0	90.20±32.6	115.32±32.7	0.033
CKD-EPI	124.34±23.8	107.10±33.3	128.26±19.5	0.010
**Obesity**				<0.001
Yes	25(7.7)	2(1.5)	23(11.8)	
No	301(92.3)	129(98.5)	172(88.2)	

Staging of kidney function and prevalence of chronic kidney disease

Using CKD-EPI estimator, about 23 (7.05 %) of the participants had stage 1 and stage 2 CKD. Whereas, only 1 (0.3 %) of them developed chronic renal failure (stage 3a-5). The overall prevalence of CKD defined as proteinuria of ≥+1 and/or GFR<60 was 7.4 % ((95% confidence interval (CI) [4.6-10.4]).

Using CG- estimator, about 21 (6.4%) of the participants had stage 1 and stage 2 CKD. Whereas, only 5 (1.5%) of them developed chronic renal failure (stage 3a-5). The overall prevalence of CKD using CG-estimator and defined as proteinuria of ≥+1 and/or GFR <60 was 8.0% ((95% confidence interval (CI) [5.2-11.0]). whereas, using MDRD- estimator, about 23 (7.0 %) of the participants had stage 1 and 2 CKD. Only 0.3% of them developed chronic renal failure (stage 3a-5). The overall prevalence of CKD Using MDRD-estimator and defined as proteinuria of ≥+1and/or GFR<60 was 7.4 % ((95% confidence interval (CI) [4.6-10.1]) as showed in Table 5.

Table 5. Stages of kidney functions using the three eGFR estimators in Sheka zone, Southwest Ethiopia, 2018.

**Stages**	**GFR estimation**	**CKD-Epi n (%)**	**CG n (%)**	**MDRD n (%)**
1	≥ 90, with proteinuria (≥+1)	19(5.8)	17(5.2)	20(6.1)
2	60–89, with proteinuria (≥+1)	4(1.2)	4(1.2)	3(0.9)
3a	45–59.9, with or without proteinuria (≥+1)	1(0.3)	4(1.2)	1(0.3)
3b	30–44.9, with or without proteinuria (≥+1)	0(0)	1(0.3)	0(0)
4	15–29.9, with or without proteinuria (≥+1)	0(0)	0(0)	0(0)
5	< 15, with or without proteinuria (≥+1)	0(0)	0(0)	0(0)
	Overall CKD	24(7.4)	26(8.0)	24(7.4)

Bivariate and multivariable analysis of associated factors

As the outcome variable is dictums, we applied logistic regression model. Before applying the model, the assumptions of logistic regression were checked. The linear relationship between continuous independent variables (age and income) and their logit transformation were checked. We found that there was no linear relationship between age and income and their log

transformations. As a result, we included age and income as categorical variables in the model. We also investigated the multicollinearity between independent variables using the variance inflation factor. A significant multicollinearity was not identified. We used three separate logistic regression models for three common GFR estimators. However, the models explained only up 41% (Nagelkerke R^2) of the variance in the CKD. The Hosmer & Lemeshow test of the three models was not significant (p>0.05) which suggests good model fitness.

Using all the three GFR estimators, age, hypertension, heart disease, central obesity and use of over the counter NSAIDs drugs regularly were significantly associated with CKD in the Bivariate analysis (p<0.05). In the full adjusted model, only hypertension was significantly associated with CKD using CKD-EPI & MDRD- estimators. On the other hand, age was significantly associated with CKD using CG- estimator in the full adjusted model. Using CKD-EPI & MDRD- estimators, participants who had hypertension were nearly 2.61 times more likely to have CKD than those who didn't have hypertension (p<0.001). Using CG- estimator, participants whose age ≥40 were 3.187 times more likely to exhibit CKD than whose age was 18-39 years (p=0.005). However, central obesity and age showed borderline significant using CKD-EPI & MDRD estimators described in Tables 6-8.

Table 6. Factors associated with CKD in age and sex adjusted logistic regressions using CKD-EPI estimator in Sheka Zone, Southwest Ethiopia, 2018.

**Variables**	**Chronic Kidney Disease**		**COR with 95% CI**	**P value**	**AOR with 95% CI**
**Socio-demographic characteristics**	**Yes (n, %)**	**No (n, %)**			
**Sex**					
Male	6(25.0)	125(41.4)	1		1
Female	18(75.0)	177(58.6)	2.12(0.82-5.49)	0.122	1.031(0.295-3.598)
**Age**					
18-39	13(54.2)	228(75.5)	1		

≥40	11(45.8)	74(24.5)	2.607(1.120-6.067)	0.026	2.347(0.902-6.109)
**Education status**					
Not attended formal education	1(4.2)	52(17.2)	0.642(0.277-1.492)	0.303	
Attended formal education	23(95.8)	250(82.8)	1		
**Marital status**					
Married	21(87.5)	246(81.5)	1		
Others	3(12.5)	56(18.5)	0.628(0.181-2.177)	0.463	
**Religion**					
Christian	17(70.8)	225(74.5)	1		
Muslim	7(29.2)	77(25.5)	1.203(0.481-3.012)	0.693	
**Occupation**					
Employed*	4(7.5)	49(92.5)			
Farming	2(3.6)	54(96.4)	0.454(0.080-2.587)	0.374	
Merchant	4(8.2)	45(91.8)	1.089(0.257-4.613)		
Housewife	11(10.6)	93(89.4)	1.449(0.438-4.789)		
Students	2(4.8)	40(95.2)	0.613(0.107-3.518)		
Others**	1(4.5)	21(95.5)	0.583(0.061-5.535)		
**Ethnicity**					
Sheka (natives)	9(37.5)	77(25.5)	1.753(0.737-4.168)	0.204	
Others***	15(62.5)	225(74.5)	1		
**Monthly income**					
<2000EB	9(45.0)	104(42.8)	1		
≥2000EB	11(55.0)	139(57.2)	0.914(0.366-2.287)	0.848	
**Main source of water for drinking**					
Wall/spring	11(45.8)	144(47.7)	0.928(0.403-2.138)	0.861	
Pip water	13(54.2)	158(52.3)	1		
**Lifestyle/behavioral characteristics**					
**Ever drink alcohol**					

Yes	1(4.2)	45(14.9)	0.248(0.033-1.885)	0.178	0.269(0.033-2.176)
No	23(95.8)	257(85.1)	1		
**Ever smoke cigarette**					
Yes	1(4.2)	16<br>5.3%	0.777(0.099-6.125)	0.811	
No	23(95.8)	286(94.7)	1		
**Smoking year**					
<10 years	0(0.0)	13(81.3)	≅0	.999	
≥10 years	1(100.0)	3(18.8)	1		
**Herbal supplement/ medicine**					
Yes	2(8.3)	8(2.6)	3.341(0.669-16.696)	0.142	0.997(0.150-6.621
No	22(91.7)	294(97.4)	1		
**Use of over the counter NSAIDs drugs regularly**					
Yes	7(29.2)	36(11.9)	3.042(1.181-7.840)	0.021	2.219(0.760-6.483
No	17(70.8)	266(88.1)	1		
**Vigorous exercise /activity**					
Yes	2(8.3)	53(17.5)	0.427(0.097-1.872	0.259	
No	22(91.7)	249(82.5)	1		
**Moderate exercise/activity**					
Yes	9(37.5)	81(26.8)	1.637(0.689-3.887)	0.264	
No	15(62.5)	221(73.2)	1		
**Eat canned/processed foods**					
Never	13(54.2)	180(59.6)	1		
Yes (occasionally and often)	11(45.8)	122(40.4)	1.248(0.542-2.878)	0.603	
**Added salt to food**					
Never	1(4.2)	17(5.6)	0.871(0.109-6.970)		0.878(0.096-8.007)
Occasionally	7(29.2)	48(15.9)	2.160(0.843-5.534)	0.109	1.593(0.546-4.647
With each meal	16(66.7)	237(78.5)	1		
**Daily water intake**					
<2 letters per day	11(45.8)	100(33.1)	1.709(0.739-3.951)	0.210	
≥ 2 letters per day	13(54.2)	202(66.9)	1		
**Co-morbidities/clinical characteristics**					

**Hypertension**					
Yes	12(50.0)	59(19.5)	4.119(1.762-9.629)	0.001	2.614(1.016-6.727
No	12(50.0)	243(80.5)	1		
**Diabetes mellitus**					
Yes	1(4.2)	10(3.3)	1.270(0.156-10.357)	0.824	
No	23(95.8)	292(96.7)	1		
**Anemia**					
Yes	6(25.0)	60(19.9)	1.344(0.512-3.533	0.548	
No	18(75.0)	242(80.1)	1		
**Heart Disease***					
Yes	3(12.5)	9(3.0)	4.651(1.170-18.479)	0.029	2.539(0.456-14.142
No	21(87.5)	293(97.0)	1		
**Kidney stone**					
Yes	4(16.7)	30(9.9)	1.813(0.581-5.657)	0.305	
No	20(83.3)	272(90.1)	1		
**Central obesity**					
Yes	13(54.2)	69(22.8)	3.991(1.711-9.306)	0.001	2.352(0.770-7.188
No	11(45.8)	233(77.2)	1		
**Nutritional status based on BMI**					
Under weight	0(0.0)	26(8.8)	≅0		
Normal weight	15(62.5)	175(58.9)	0.986(0.212-4.589)		
Overweight	7(29.2)	73(24.6)	1.103(0.214-5.684)	0.907	
Obese	2(8.3)	23(7.7)	1		

Table 7. Factors associated with CKD in age and sex adjusted logistic regressions using MDRD estimator in Sheka Zone, Southwest Ethiopia, 2018.

**Variables**	**Chronic Kidney Disease**		**COR with 95% CI**	**P value**	**AOR with 95% CI**
**Socio-demographic characteristics**	**Yes (n, %)**	**No (n, %)**			
**Sex**					
Male	6(25.0)	125(41.4)	1		1
Female	18(75.0)	177(58.6)	2.119(0.818-5.489)	0.122	1.031(0.295-3.598)

**Age**					
18-39	13(54.2)	228(75.5)	1		
≥40	11(45.8)	74(24.5)	2.607(1.120-6.067)	0.026	2.347(0.902-6.109)
**Education status**					
Not attended formal education	1(4.2)	52(17.2)	0.209(0.028-1.582)	0.303	
Attended formal education	23(95.8)	250(82.8)	1		
**Marital status**					
Married	21(87.5)	246(81.5)	1		
Others	3(12.5)	56(18.5)	0.628(0.181-2.177)	0.463	
**Religion**					
Christian	17(70.8)	225(74.5)	1		
Muslim	7(29.2)	77(25.5)	1.203(0.481-3.012)	0.693	
**Occupation**					
Employed*	4(16.7)	49(16.2)			
Farming	2(8.3)	54(17.9)	0.454(0.080-2.587)	0.374	
Merchant	4(16.7)	45(14.9)	1.089(0.257-4.613)		
Housewife	11(45.8)	93(30.8)	1.449(0.438-4.789)		
Students	2(8.3)	40(13.2)	0.613(0.107-3.518)		
Others**	1(4.2)	21(7.0)	0.583(0.061-5.535)		
**Ethnicity**					
Sheka(natives)	9(37.5)	77(25.5)	1.753(0.737-4.168)	0.204	
Others***	15(62.5)	225(74.5)	1		1
**Monthly income**					
<2000EB	9(45.0)	104(42.8)	1		1
≥2000EB	11(55.0)	139(57.2)	0.914(0.366-2.287)	0.848	
**Main source of water for drinking**					
Wall/spring	11(45.8)	144(47.7)	0.928(0.403-2.138)	0.861	
Pip water	13(54.2)	158(52.3)	1		1
**Lifestyle/behavioral characteristics**					
**Ever drink alcohol**					
Yes	1(4.2)	45(14.9)	0.248(0.033-1.885)	0.178	0.269(0.033-2.176)
No	23(95.8)	257(85.1)	1		1
**Ever smoke cigarette**					

Yes	1(4.2)	16(5.3)	0.777(0.099-6.125)	0.811	
No	23(95.8)	286(94.7)	1		
**Smoking year**					
<10 years	0(0.0)	13(81.3)	≅0	0.999	
≥10 years	1(100.0)	3(18.8)	1		
**Herbal supplements medicine**					
Yes	2(8.3)	8(2.6)	3.341(0.669-16.696)	0.142	0.997(0.150-6.621)
No	22(91.7)	294(97.4)	1		1
**Use of over the counter NSAIDs drugs regularly**					
Yes	7(29.2)	36(11.9)	3.042(1.181-7.840)	0.021	2.219(0.760-6.483)
No	17(70.8)	266(88.1)	1		1
**Vigorous exercise /activity**					
Yes	2(8.3)	53(17.5)	0.427(0.097-1.872)	0.259	
No	22(91.7)	249(82.5)	1		
**Moderate exercise/activity**					
Yes	9(37.5)	81(26.8)	1.637(0.689-3.887)	0.264	
No	15(62.5)	221(73.2)	1		
**Eat canned/processed foods**					
Never	13(54.2)	180(59.6)	1		
Yes (occasionally and often)	11(45.8)	122(40.4)	1.248(0.542-2.878)	0.603	
**Added salt to food**					
Never	1(4.2)	17(5.6)	0.871(0.109-6.970)		0.878(0.096-8.007)
Occasionally	7(29.2)	48(15.9)	2.160(0.843-5.534)	0.109	1.593(0.546-4.647)
With each meal	16(66.7)	237(78.5)	1		1
**Daily water intake**					
<2 letters per day	11(45.8)	100(33.1)	1.709(0.739-3.951)	0.210	
≥ 2 letters per day	13(54.2)	202(66.9)	1		
**Co-morbidities/clinical characteristics**					
**Hypertension**					
Yes	12(50.0)	59(19.5)	4.119(1.762-9.629)	0.001	2.614(1.016-6.727)
No	12(50.0)	243(80.5)	1		1
**Diabetes**					
Yes	1(4.2)	10(3.3)	1.270(0.156-10.357)	0.824	
No	23(95.8)	292(96.7)	1		
**Anemia**					

Yes	6(25.0)	60(19.9)	1.344(0.512-3.533)	0.548	
No	18(75.0)	242(80.1)	1		
**Heart Disease***					
Yes	3(12.5)	9(3.0)	4.651(1.170-18.479)	0.029	2.539(0.456-14.142)
No	21(87.5)	293(97.0)	1		1
**Kidney stone**					
Yes	4(16.7)	30(9.9)	1.813(0.581-5.657)	0.305	
No	20(83.3)	272(90.1)	1		
**Central Obesity**					
Yes(obese)	13(54.2)	69(22.8)	3.991(1.711-9.306)	0.001	2.352(0.770-7.188)
No (not obese)	11(45.8)	233(77.2)	1		1
**Nutritional status based on BMI**					
Under weight	0(0.0)	26(8.8)	≅0		
Normal weight	15(62.5)	175(58.9)	0.986(0.212-4.589)		
Overweight	7(29.2)	73(24.6)	1.103(0.214-5.684)	0.907	
Obese	2(8.3)	23(7.7)	1		

Table 8. Factors associated with CKD in age and sex adjusted logistic regressions using CG estimator in Sheka Zone, Southwest Ethiopia, 2018.

**Variables**	**Chronic Kidney Disease**		**Unadjusted**	**P-value**	**Adjusted**
**Socio-demographic characteristics**	**Yes (n, %)**	**No n, %)**			
**Sex**					
Male	6(23.1)	125(41.7)	1		1
Female	20(76.9)	175(58.3)	2.381(0.929-6.100)	0.071	1.340(0.404-4.442)
**Age**					
18-39	13(50.0)	228(76.0)	1		
≥40	13(50.0)	72(24.0)	3.167(1.404-7.141)	0.005	3.187(1.281-7.926)
**Education status**					
Not attended formal education	2(7.7)	51(17.0)	0.407(0.093-1.776)	0.232	
Attended formal education	24(92.3)	249(83.0)	1		1
**Marital status**					
Married	23(88.5)	244(81.3)	1		1

Others	3(11.5)	56(18.7)	0.568(0.165-1.959)	0.371	
**Religion**					
Christian	18(69.2)	224(74.7)	1		1
Muslim	8(30.8)	76(25.3)	1.310(0.547-3.135)	0.544	
**Occupation**					
Employed*	4(15.4)	49(16.3)	1		
Farming	3(11.5)	53(17.7)	0.693(0.148-3.256)		
Merchant	4(15.4)	45(15.0)	1.089(0.257-4.613)	0.438	
Housewife	12(46.2)	92(30.7)	1.598(0.489-5.218)		
Students	2(7.7)	40(13.3)	0.613(0.107-3.518)		
Others**	1(3.8)	21(7.0)	0.583(0.061-5.535)		
**Ethnicity**					
Sheka (natives)	10(38.5)	76(25.3)	1.842(0.802-4.232)	0.150	
Others***	16(61.5)	224(74.7)	1		1
**Monthly income**					
<2000EB	11(50.0)	102(42.3)	1		1
≥2000EB	11(50.0)	139(57.7)	0.734(0.306-1.758)	0.488	
**Main source of water for drinking**					
Wall/spring	13(50.0)	142(47.3)	1.113(0.499-2.480)	0.794	
Pip water	13(50.0)	158(52.7)	1		1
**Lifestyle/behavioral characteristics**					
**Ever drink alcohol**					
Yes	1(3.8)	45(15.0)	0.227(0.030-1.715)	0.151	0.253(0.032-2.025)
No	25(96.2)	255(85.0)	1		
**Ever smoke cigarette**					
Yes	1(3.8)	16(5.3)	0.710(0.090-5.578)	0.745	
No	25(96.2)	284(94.7)	1		
**Smoking year**					
<10 years	0(0.0)	13(81.3)	≅0	0.999	
≥10 years	1(100.0)	3(18.8)	1		
**Herbal supplements /medicine**					
Yes	2(7.7)	8(2.7)	3.042(0.611-15.132)	0.174	1.106(0.167-7.342)
No	24(92.3)	292(97.3)	1		

**Use of over the counter NSAIDs drugs regularly**					
Yes	7(26.9)	36(12.0)	2.702(1.062-6.875)	0.037	1.937(0.673-5.576)
No	19(73.1)	264(88.0)	1		
**Vigorous exercise /activity**					
Yes	2(7.7)	53(17.7)	0.388(0.089-1.694)	0.208	
No	24(92.3)	247(82.3)	1		
**Moderate exercise/activity**					
Yes	10(38.5)	80(26.7)	1.719(0.749-3.944)	0.201	
No	16(61.5)	220(73.3)	1		
**Eat canned/processed foods**					
Never	15(57.7)	178(59.3)	1		
Yes (occasionally and often)	11(42.3)	122(40.7)	1.070(0.475-2.409)	0.870	
**Added salt to food**					
Never	1(3.8)	17(5.7)	0.768(0.097-6.104)		0.716(0.078-6.543)
Occasionally	7(26.9)	48(16.0)	1.904(0.754-4.809)	0.173	1.471(0.511-4.233)
With each meal	18(69.2)	235(78.3)	1		
**Daily water intake**					
<2 letters per day	11(42.3)	100(33.3)	1.467(0.650-3.311)	0.357	
≥ 2 letters per day	15(57.7)	200(66.7)	1		
**Co-morbidities/clinical**					
**Hypertension**					
Yes	12(46.2)	59(19.7)	3.501(1.539-7.965)	0.003	2.105(0.836-5.299)
No	14(53.8)	241(80.3)	1		
**Diabetes**					
Yes	1(3.8)	10(3.3)	1.160(0.143-9.433)	0.890	
No	25(96.2)	290(96.7)	1		
**Anemia**					
Yes	6(23.1)	60(20.0)	1.200(0.462-3.119)	0.708	
No	20(76.9)	240(80.0)	1		
**Heart Disease***					
Yes	3(11.5)	9(3.0)	4.217(1.068-16.661)	0.040	2.555(0.462-14.133)
No	23(88.5)	291(97.0)	1		
**Kidney stone**					
Yes	4(15.4)	30(10.0)	1.636(0.529-5.066)	0.393	
No	22(84.6)	270(90.0)	1		
**Central Obesity**					

Yes	14(53.8)	68(22.7)	3.980(1.758-9.011)	0.001	2.147(0.748-6.162)
No	12(46.2)	232(77.3)	1		
**Nutritional status based on BMI**					
Under weight	1(3.8)	25(8.5)	0.460(0.039-5.418)	0.537	
Normal weight	15(57.7)	175(59.3)	0.986(0.212-4.589)		
Overweight	8(30.8)	72(24.4)	1.278(0.253-6.451)		
Obese	2(7.7)	23(7.8)	1		

Discussion

In the current study, we assessed the prevalence and associated risk factors of chronic kidney diseases among the urban adult population in Sheka zone using the three commonest estimators of kidney functions. Over 7% of urban adults had CKD regardless of whether CKD-EPI, CG or MDRD GFR estimators were used. This finding is comparable with the prevalence of CKD reported in other sub-Saharan African counties; 10.4% in Nigeria [14] , 12.4% in Kinshasa Congo [15], 10% in Cameroon [10], and 7% in Tanzania with 15.2% in the urban population[16]. The prevalence of CKD was slightly higher using the CG GFR estimator. It has been documented that CG overestimates the true prevalence of CKD[10,16]. But, a similar prevalence of CKD was estimated using CKD-EPI and MDRD estimators. This may indicate that either CKD-EPI or MDRD can be used to estimate GFR in this study area. Other study findings, for instance, in Southern Africa and Ghana also showed that there is a high agreement between these two GFR estimators [17,18].

Using NKF/KDOQI staging, we found that most of the participants were in stage I & II. The finding is in line with several study findings in developing countries [9-11]. People within the early stage of CKD may not aware of their status. In fact, several studies showed that the majority of

CKD patients don't aware their kidney problems [15,19,20]. Identification of people in the early stage of CKD is important. This is because the cardiovascular risks associated with stage I & II is nearly equal to that of stage III [21].

After adjusting socio-demographic, lifestyle and co-morbidities related variables, only hypertension and advanced age were found to be significantly associated with CKD using CKD-EPI/MDRD & CG estimators respectively. However, more of the increased prevalence of CKD in this study area seems to be unexplained. In line with our study finding, a recent study finding in Tanzania and Uganda showed that the majority of the increase in the risk of CKD was not explained [9,11]. Unexplained risk factors of CKD in this study area may suggest that other non-traditional risk factors might contribute to the high burden of CKD. Some study findings in another part of the world showed that nontraditional risk factors such as heavy metals and agro-chemicals may contribute to CKD in tropical and semi-tropical areas [22,23]. Some other studies suggested that infectious diseases such as HIV/AIDS, Schistosomiasis and Leishmaniasis are also associated with CKD [24-26]. However, as a limitation of this study, we didn't control the confounding effect of these risk factors of CKD. Large-scale epidemiological studies are needed to better understand the specific risk factors of CKD and, to examine the potential but unmeasured above risk factors.

As documented in the broad literature [7,10,11,19], advanced age is a well-known risk factor of CKD. As age increases, there may be salt sensitization, hardening of arteries and scarring of the tiny blood vessels in the kidney. This may, in turn, enhance protein excretion and decrease GFR [27-29]. However, as many of the adult participants in the current study didn't have a birth certificate, they may underestimate their age. Underestimation of age could lead to overestimation of GFR using any of the methods used to calculate GFR. This may in turn underestimate the true prevalence of CKD in this study area.

In the current study, among the traditional risk factors of CKD, hypertension was found to be significantly associated with CKD. This is in line with several community-based study findings [10,11,14,20]. Hypertension may elevate intraglomerular pressure which results in glomerulosclerosis and eventual protein trafficking [30,31]. However, as a limitation of this study (chicken egg dilemma), it is hard to distinguish whether hypertension causes CKD or CKD causes hypertension. An increased risk of CKD among hypertensive participants may warrant urban adults to adhere to treatment and control their blood pressures. Studies have shown that interventions that decrease blood pressure levels in patients with proteinuria delay progression to CKD [32,33].

Co-morbidity related factors such as diabetes, anemia, kidney stone; heart disease, central obesity and nutritional status measured by BMI were not significantly associated with CKD. However, central obesity showed a borderline significant. It has been documented that waist circumference or waist to hip ratio predicts cardiovascular risks than BMI [34]. The low prevalence of the above-mentioned co-morbidities in our participants makes any comment of their association difficult to ascertain.

In the current study, behavioral factors such as alcohol intake and cigarette smoking were not significantly associated with CKD. This is in line with several study findings [14,35,36]. Alcohol intake and cigarette smoking are often considered anti-social habits in Ethiopia so people may give a negative response. This may, in turn, nullify the true association between alcohol intake, cigarette smoking and, CKD.

Overall, in this study area, CKD is significantly associated with age ≥40 years old and hypertension. Early screening of high-risk populations (elderly and hypertensive peoples in the case of this study area) may help to diagnose people at the early stage of CKD. This is very

important as Ethiopian health care institutions may not be able to support the economic burden of providing dialysis and renal replacement therapy to all patients. Dialysis and renal replacement therapy are not free in Ethiopia. This may result catastrophic out of the pocket expenditure. Thus, early screening of high-risk populations to CKD is important to minimize complications and out of pocket expenditures.

This study was the first community-based assessment of CKD in Southwest Ethiopia using the three common GFR estimators. The finding of the study will provide information about the urban prevalence of CKD in Sheka zone. However, this study has several limitations. The cross-sectional nature of this study restricted us to infer causality. The common GFR measures used in this study were not validated in Ethiopia. The absence of reliable and validated measures of kidney function tests has made difficult the estimation of CKD in Africa [16,18]. This study exclusively focused on the urban population. Thus, the estimated CKD may not reflect the prevalence and risk factors of rural communities. Because of security and financial restrictions, we didn’t make a three months control of positive findings as suggested by the KDIGO guideline. Some risk factors identified elsewhere such as heavy metals, agrochemicals, Schistosomiasis, Leishmaniasis and HIV [22-26] were not investigated in this study.

Conclusions

The prevalence of CKD was comparable with other sub-Saharan African countries. Only hypertension and age ≥ years old were significantly associated with CKD. More of the increased prevalence of CKD in the current study remained unexplained and deserves further study. Policymakers and programmers need to design strategies and encourage high-risk populations to be screened as early as possible. It is better if clinicians should also consider kidney function marker tests for hypertensive and patients older than 40 years old.

Acknowledgments

Special gratitude goes to Mizan-Tepi University for providing funds for this study. The authors would like to acknowledge all the data collectors, supervisors and study participants.

References

1. Jha V, Garcia-Garcia G, Iseki K, Li Z, Naicker S, et al. (2013) Chronic kidney disease: global dimension and perspectives. The Lancet 382: 260-272.
2. Hill NR, Fatoba ST, Oke JL, Hirst JA, O'Callaghan CA, et al. (2016) Global prevalence of chronic kidney disease–a systematic review and meta-analysis. PloS one 11.
3. Yirsaw BD (2012) Letter to the Editor-Chronic kidney disease in sub-Saharan Africa: Hypothesis for research demand. Annals of African medicine 11: 119-120.
4. Baynes HW, Negash M, Geremew D, Getaneh Z (2019) Chronic Kidney Disease among Human Immunodeficiency Virus Positive Patients on Antiretroviral Therapy in Sub-Saharan Africa: A Systematic Review and Meta-analysis. Saudi Journal of Kidney Diseases and Transplantation 30: 1190-1200.
5. Global HIV and AIDS Statistics. 2018 Global HIV Statistics. Available from: http://www.avert.org/global-hiv-and-aids-statistics. (Accessed on April 11, 2020).
6. World Health Organization. Noncommunicable Dseases Country Profiles 2018. WHO, 2018 (Accessed on April 10, 2020).
7. Fiseha T, Kassim M, Yemane T (2014) Chronic kidney disease and underdiagnosis of renal insufficiency among diabetic patients attending a hospital in Southern Ethiopia. BMC nephrology 15: 198.
8. Mekuria Y, Yilma D, Mekonnen Z, Kassa T, Gedefaw L (2016) Renal Function Impairment and Associated Factors among HAART Naïve and Experienced Adult HIV Positive Individuals in Southwest Ethiopia: A Comparative Cross Sectional Study. PloS one 11.
9. Stanifer JW, Maro V, Egger J, Karia F, Thielman N, et al. (2015) The epidemiology of chronic kidney disease in Northern Tanzania: a population-based survey. PloS one 10.

10. Kaze FF, Halle M-P, Mopa HT, Ashuntantang G, Fouda H, et al. (2015) Prevalence and risk factors of chronic kidney disease in urban adult Cameroonians according to three common estimators of the glomerular filtration rate: a cross-sectional study. BMC nephrology 16: 96.

11. Kalyesubula R, Nankabirwa JI, Ssinabulya I, Siddharthan T, Kayima J, et al. (2017) Kidney disease in Uganda: a community based study. BMC nephrology 18: 116.

12. Coudène P, Marson B, Badiou S, Flavier S, Anelli S, et al. (2005) Evaluation of the ABX Pentra 400: a newly available clinical chemistry analyser. Clinical Chemistry and Laboratory Medicine (CCLM) 43: 782-792.

13. Levey AS, Inker LA (2014) Definition and staging of chronic kidney disease in adults. V: UpToDate, Post TW (Ed), UpToDate, Waltham, MA (Pridobljeno 5 Sep 2014).

14. Afolabi MO, Abioye-Kuteyi AE, Arogundade FA, Bello IS (2009) Prevalence of chronic kidney disease in a Nigerian family practice population. South African Family Practice 51.

15. Sumaili EK, Krzesinski J-M, Zinga CV, Cohen EP, Delanaye P, et al. (2008) Prevalence of chronic kidney disease in Kinshasa: results of a pilot study from the Democratic Republic of Congo. Nephrology Dialysis Transplantation 24: 117-122.

16. Stanifer JW, Jing B, Tolan S, Helmke N, Mukerjee R, et al. (2014) The epidemiology of chronic kidney disease in sub-Saharan Africa: a systematic review and meta-analysis. The Lancet Global Health 2: e174-e181.

17. Matsha TE, Yako YY, Rensburg MA, Hassan MS, Kengne AP, et al. (2013) Chronic kidney diseases in mixed ancestry south African populations: prevalence, determinants and concordance between kidney function estimators. BMC nephrology 14: 75.

18. Eastwood JB, Kerry SM, Plange-Rhule J, Micah FB, Antwi S, et al. (2010) Assessment of GFR by four methods in adults in Ashanti, Ghana: the need for an eGFR equation for lean African populations. Nephrology Dialysis Transplantation 25: 2178-2187.

19. Abu-Aisha H, Elhassan A, Khamis A, Abu-Elmaali A (2009) Chronic kidney disease in police forces households in Khartoum, Sudan: pilot report. Arab Journal of Nephrology and Transplantation 2: 21-26.

20. HWANG SJ, TSAI JC, CHEN HC (2010) Epidemiology, impact and preventive care of chronic kidney disease in Taiwan. Nephrology 15: 3-9.

21. Glassock RJ, Winearls C (2008) Screening for CKD with eGFR: doubts and dangers. Clinical Journal of the American Society of Nephrology 3: 1563-1568.

22. Lozier M, Turcios-Ruiz RM, Noonan G, Ordunez P (2016) Chronic kidney disease of nontraditional etiology in Central America: a provisional epidemiologic case definition for surveillance and epidemiologic studies. Revista Panamericana de Salud Pública 40: 294-300.

23. Chapman E, Haby MM, Illanes E, Sanchez-Viamonte J, Elias V, et al. (2019) Risk factors for chronic kidney disease of non-traditional causes: a systematic review.

24. Wyatt CM, Meliambro K, Klotman PE (2012) Recent progress in HIV-associated nephropathy. Annual review of medicine 63: 147-159.

25. Wyatt CM, Klotman PE, D'Agati VD. HIV-associated nephropathy: clinical presentation, pathology, and epidemiology in the era of antiretroviral therapy; 2008. Elsevier. pp. 513-522.

26. da Silva Junior GB, Duarte DB, Barros EJG, Daher EDF (2013) Schistosomiasis-associated kidney disease: A review. Asian Pacific Journal of Tropical Disease 3: 79-84.

27. Iseki K, Iseki C, Ikemiya Y, Kinjo K, Takishita S (2007) Risk of developing low glomerular filtration rate or elevated serum creatinine in a screened cohort in Okinawa, Japan. Hypertension research 30: 167.

28. Shannon R, Minaker K, Rowe J (1984) The influence of age on water balance in man. Semin Nephrol 4: 346-352.

29. Poulter N, Khaw K, Hopwood B, Mugambi M, Peart W, et al. (1984) Salt and blood pressure in various populations. Journal of cardiovascular pharmacology 6: S197-203.

30. Grimm Jr RH, Svendsen KH, Kasiske B, Keane WF, Wahl MM (1997) Proteinuria is a risk factor for mortality over 10 years of follow-up. Kidney International Supplement.

31. Bianchi S, Bigazzi R, Campese VM (1999) Microalbuminuria in essential hypertension: significance, pathophysiology, and therapeutic implications. American journal of kidney diseases 34: 973-995.

32. Jafar TH, Stark PC, Schmid CH, Landa M, Maschio G, et al. (2003) Progression of chronic kidney disease: the role of blood pressure control, proteinuria, and angiotensin-converting enzyme inhibition: a patient-level meta-analysis. Annals of internal medicine 139: 244-252.

33. Peterson JC, Adler S, Burkart JM, Greene T, Hebert LA, et al. (1995) Blood pressure control, proteinuria, and the progression of renal disease: the Modification of Diet in Renal Disease Study. Annals of internal medicine 123: 754-762.

34. Taylor EN, Stampfer MJ, Curhan GC (2005) Obesity, weight gain, and the risk of kidney stones. Jama 293: 455-462.

35. Vupputuri S, Sandler DP (2003) Lifestyle risk factors and chronic kidney disease. Annals of epidemiology 13: 712-720.

36. Stengel B, Tarver–Carr ME, Powe NR, Eberhardt MS, Brancati FL (2003) Lifestyle factors, obesity and the risk of chronic kidney disease. Epidemiology 14: 479-487.

Supporting information

S1 File. Questionnaire in English and local language (Amharic).

Fig 1. Definitions and stages of Chronic Kidney Disease

Click here to access/download;Figure;Figure 1.tif

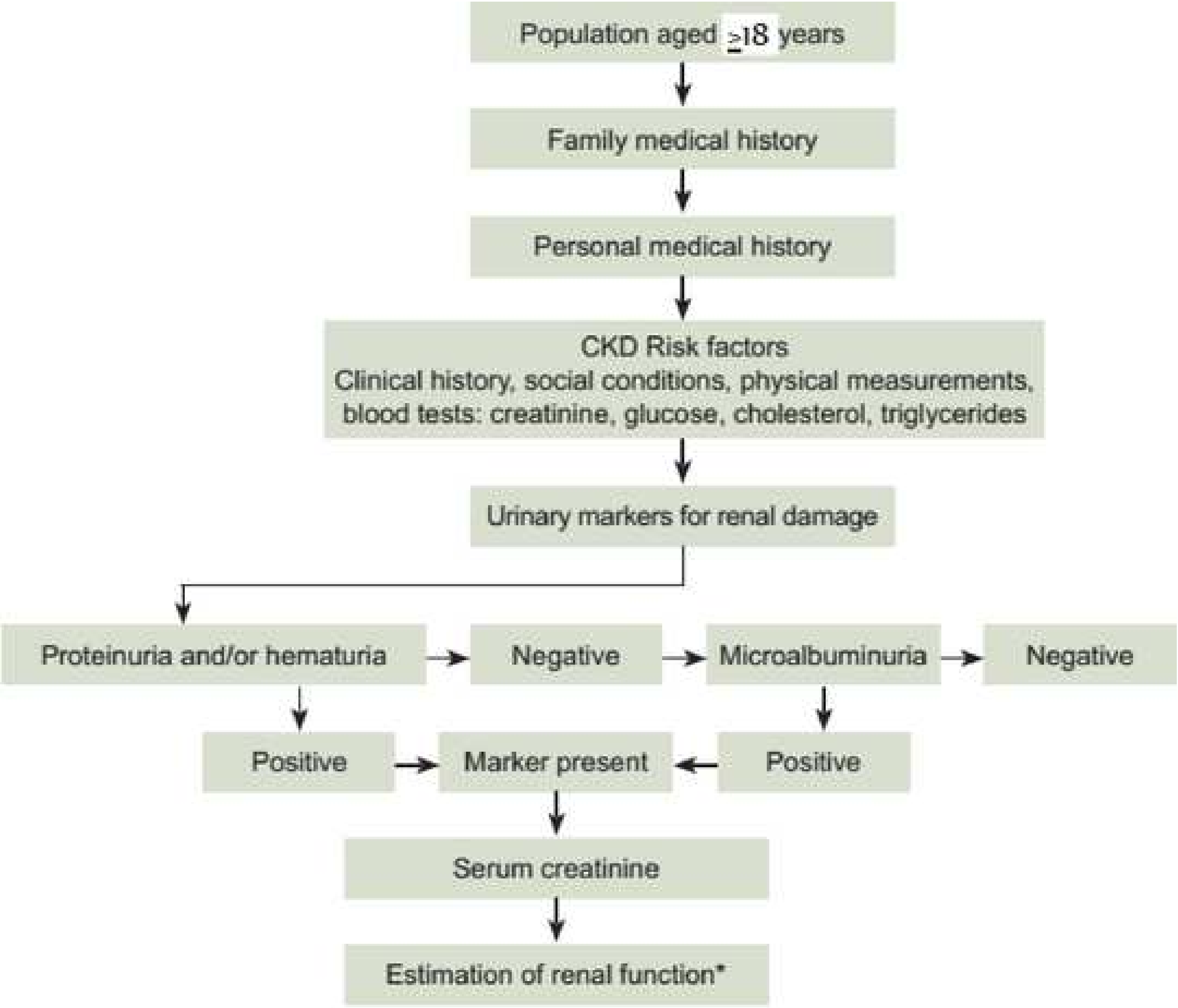

	CKD Stages	Description	GFR (mL/min/1.73 m²)
Urinary markers for renal damage	1	Kidney damage with normal or increased GFR	≥90
	2	Kidney damage with mildly decreased GFR	60-89
Chronic renal failure	3a	Kidney damage with moderately decreased GFR	45-59
	3b		30-44
	4	Severely decreased GFR	15-29
	5	ESRD	<15

Click here to access/download
Supporting Information
Questionnaire in English and local language (Amharic).pdf

Chronic Kidney Disease and Associated Factors among Adult Population in Southwest Ethiopia

Kindie Mitiku Kebede[1], Dejene Derseh Abateneh[1,2]*, Melkamu BeyeneTeferi[1], Abyot Asres[1]

[1]College of Medicine and Health Sciences, Mizan Tepi University, Mizan Teferi, Ethiopia.

[2]Menelik II College of Medicine and Health Sciences, Kotebe Metropolitan University, Addis Ababa, Ethiopia.

***Corresponding author:**

Mailing address: Kotebe Metropolitan University, Menelik II College of Medicine and Health Sciences, Department of Medical Laboratory Sciences, Addis Ababa, Ethiopia. E-mail: dejenieh@gmail.com, P.O.BoX: 3268. Tel: +251920514158.

Abstract

In Ethiopia, data on the burden and determinates of chronic kidney disease (CKD) in the general population is limited. This community-based study was conducted to assess the burden and associated factors of CKD among adults in Southwest Ethiopia. The study was conducted from August 23, 2018-October 16, 2018. Study participants were selected using a random sampling method. A structured questionnaire was used to collect relevant data. Blood pressure and anthropometric measurements were made following standard procedures. About 5 ml of urine sample was collected and the dipstick test was performed immediately. A blood sample of 3-5ml was collected for serum creatinine and blood glucose level determination. The three commonest estimators of glomerular filtration rate and the National Kidney Foundation Kidney Disease Outcomes Quality Initiative were used to define and stage CKD. Data were analyzed using SPSS 21. Multivariable logistic regression was employed and p-value <0.05 was used to indicate statistically significant results. A total of 326 participants with a mean age of 39.9(SD±11.2) years were enrolled in the study. The proportions of female participants (59.8%) were relatively higher

than male participants (40.2%). The mean eGFR using CKD-EPI, CG and MDRD was 124.34 (SD±23.8), 110.67(SD±33.0) and 131.29 (SD±32.5) respectively. The prevalence of CKD was 7.4% using CKD-EPI & MDRD and 8% using CG. Similar finding using CKD-EPI & MDRD may indicate that either CKD-EPI or MDRD can be used to estimate GFR in this study area. In the age and sex-adjusted logistic regression model, hypertension was significantly associated with CKD using CKD-EPI & MDRD and age ≥40 years old was significantly associated with CKD using CG. Behavioral characteristics and other traditional risk factors were not significantly associated with CKD in the current study. The prevalence of CKD was comparable with other sub-Saharan African countries. Only hypertension and age ≥40 years old were significantly associated with CKD. More of the increased prevalence of CKD in the current study remained unexplained and deserves further study.

Keywords: Chronic kidney disease, Associated factors, Adult, Ethiopia

Introduction

Chronic kidney disease (CKD) is among Non-communicable diseases (NCD) which cause significant morbidity and mortality globally [1]. A recent systematic review and meta-analysis estimated that the global pooled prevalence of CKD ranges from 11–13% and 13.9% prevalence in sub-Saharan Africa [2]. The epidemiology of CKD in sub-Saharan Africa is different from other regions that it mainly affects the economically productive young age group. The risk of CKD like HIV infection, hypertension, diabetes, and other infectious diseases are increasing in sub-Saharan Africa [2-5].

Although Ethiopia is among the countries that strive to achieve sustainable development goals 2016-2030, the progress towards the reduction of NCD associated deaths is limited. More than one

third of, (39%) annual deaths are associated with NCD [6]. The burden of CKD is increasing and mainly associated with poor community awareness, insufficient data, and poor healthcare infrastructure [7]. In Ethiopia, studies that assess the prevalence and associated factors of CKD are very limited. The available studies are not representative because they are taken from high-risk groups, like, diabetes and HIV patients [7,8]. A high prevalence of CKD, 18.2% and 23.8% using the Modification of Diet in Renal Disease (MDRD) and Cockcroft-Gault (C-G) equations respectively was reported among diabetic patients [7]. Owing to limited community based published reports available so far on CKD in Ethiopia, this study was conducted to assess the prevalence and associated factors of CKD among adults in Southwest Ethiopia.

Materials and Methods

Study area, study design, and participants

A community-based cross-sectional study was conducted in the Sheka zone, Southern Nation, Nationality and People Region (SNNPR), Southwest Ethiopia. It is bordered on the South by Bench Maji, on the West by the Gambella region, on the North by the Oromia region and on the East by the Kaffa zone. The administrative center of Sheka is Masha which is located approximately 610 Km Southwest of Addis Ababa. Based on the 2007 census conducted by the central statistical agency, the zone had a total population of 199,314 of whom 101,059 were men and 98,255 were women. About 34, 2227 (17.17%) were urban inhabitants.

The study was conducted from August 23, 2018 to October 16, 2018. The minimum sample size (N) was determined by using single population proportion formula [$N = (Z\ \alpha/2)^{2} P\ (1\text{-}P)\ /d^{2}$], where $Z_{\alpha/2}$ = the value under standard normal table at 95% level of confidence which is 1.96, prevalence P, 15.2% prevalence of CKD among urban adults [9], margin of error d, which was set at 4%. Including a 10% non-response rate, the final sample size was 384 adults. Permanent

residents aged ≥18 years in the households of Masha and Tepi town were included in the study. However, pregnant women and participants with acute illness with fever during data collection were excluded. Mentally disabled and those unable to give response were also excluded from the study.

Sampling procedure

A total of two urban areas of Sheka zone was selected randomly i.e. Tepi and Masha from a total of three urban areas (Tepi, Mash and Andracha). Tepi town had three kebeles, while Masha town had two kebeles during the study period. A total of 3 kebeles were randomly included in the study from a total of 5 kebeles in the selected urban areas. Then, the lists of households were taken from the administration of each kebele. All households in the selected urban kebeles with adults ≥18 years old were enumerated with the help of health extension workers to generate the sampling frame. After the generation of the sample framing, the sample size was allocated proportionally to the total household of each kebele. Finally, eligible participants were selected using a random sampling method. In the case of more than one adult population in a single household, one was selected using the lottery method. In case where eligible respondents were not available at the time of data collection, a revisit for three times was made. Respondents who were not available after three visits were considered as non-respondents.

Data collection and laboratory methods

A structured questionnaire adapted from various literatures [9-11] was used to collect data on socio-demographic, economic, behavioral and co-morbidity related characteristics of participants. Height, weight, blood pressure and waist circumference of all participants were measured by trained data collectors following standardized procedures. Blood pressure readings were performed using a digital blood pressure apparatus after the participant had remained in a sited

position for three to five minutes on his or her right arm resting on the chair. Weight, height and waist circumference were measured in light clothing without shoe. Height was taken to the nearest 0.1 cm with a portable stadiometer (Seca 274, Germany) and weight was measured to the nearest 0.1k.g on mechanical Seca (Seca761, Germany). Waist circumference was measured using tape measures on the midway between the top of the hip bone and the bottom of the ribs. Participants were instructed to put off their heavy clothes and breathe out normally before waist circumference measurements. Waist circumference measurements were taken to the nearest 0.1cm. Blood pressure and all anthropometric measurements were made twice, and their averages were used in all analysis.

About 5 ml random urine sample was collected using a clean, leak-proof urine cup for urine chemical analysis. Chemical analysis of urine specimens was performed immediately after sample collection using urine dipsticks test (Multistix® Henry Schein, Inc. https://www.henryschein.com/medical-multistix.aspx). Semi-quantitative chemical analysis of CKD markers (protein) was performed.

Blood sample of 3 - 5 ml for serum creatinine and blood glucose level determination was collected using a syringe with needle and transferred to a gel containing serum separator test tube. Blood specimens were transported to the nearest general hospital (Tepi general hospital) for serum separation. A serum was separated after the sample clotted and centrifuged at 1000 – 2000g for 10 minutes by trained laboratory technologist. Serum sample was immediately separated from the whole blood and transferred to nunc tube. The serum was kept frozen at -20 ºC until processed and then transported on ice-cooled containers to Jimma teaching and referral hospital, Ethiopia for analysis.

The creatinine and blood glucose levels were measured using the ABX pentra400 chemistry analyzer and reported in mg/dL or µmol/L (HORIBA, ABX, Japan). The ABX Pentra 400 is a multiparametric analyzer in which it has good reliability and practicability for routine and specialized clinical chemistry analyses, evaluated according to the National Committee for Clinical Laboratory Standards [12].

Variables' definition

The three common equations were used to estimate glomerular filtration rate (eGFR), and the National Kidney Foundation Kidney Disease Outcomes Quality Initiative (NKF/KDOQI) guidelines to define and stage CKD [13]. We defined a participant as having CKD if he or she had GFR below 60 ml/min/1.73 m^2 and/or had proteinuria (positive dipstick at least +1). Glomerular filtration rate classification was based on the following: stage 1: GFR > 90 with proteinuria; stage 2: GFR 60 – 89 with proteinuria; stage 3a: GFR 45 – 59; stage 3b: GFR 30 – 44; stage 4: GFR 15 – 29 and stage 5 end-stage renal disease (ESRD): GFR < 15 (Fig 1). Hypertension was defined as a systolic (SBP) ≥140 mmHg and/or a diastolic blood pressure of (DBP) ≥90 mmHg or self-reported on anti-hypertension drugs. Diabetes was defined as random blood sager >200mg/dl and/or self-reported on anti-hyperglycemic medication. A separate cut of points was used to level central obesity for male men and women: women were leveled as obese if their waist circumference was ≥88 cm and men were leveled as obese if their waist circumference ≥102 cm. The nutritional status of participants was assessed using body mass index (BMI) and leveled as follows: underweight: <18.5; normal weight: 18.5 – 24.9; overweight: 25 – 29.9 and obese: >30.

Traditional causes of CKD: Refer to the well-known causes of CKD such as hypertension, diabetes, obesity, and HIV.

Heart diseases: In this study, it means a self-reported history of heart diseases such as coronary disease (vessel surgery), or heart failure, or heart attack or stroke.

Moderate activities/exercise: Brisk walking or carrying light loads, gardening, household /domestic chores, roofing, painting for 30 minutes per day for five days a week

Vigorous exercise/activity: Carrying or lifting heavy loads, digging or construction work, running, fast cycling, aerobics, fast swimming, playing football & volleyball for 30 minutes per day for five days a week.

Fig 1. Definitions and stages of Chronic Kidney Disease [13].

Data analysis and interpretation

Data were entered into Epidata version 3.1 and exported to SPSS 21.0 for further analysis. Continuous data were presented as mean (± standard deviation) and categorical data as proportions. We used chi-square and fishers exact test for comparison of proportions and independent t-test for comparing mean differences. Logistic regression models were used to investigate the predictors of CKD. Variables with p-value of <0.2 in the bivariate analysis were included in the multivariable analysis. A p-values <0.05 were used to indicate statistically significant results.

Data quality control

Data collectors were trained and supervised during the data collection process. The questionnaire was first prepared in the English language and translated to the local language (Amharic). About 10% of the questionnaire was pretested among urban Kebeles of Sheka zone which were not included in this study before the actual study period. Completeness and consistency of the collected data were checked during data collection by the supervisors. Anthropometric equipment's were calibrated frequently for every five measurements to control fallacy. Both intra-observer and

interobserver variations were checked using SPSS version 21.0 during training of anthropometric measurement and the training was continued until an acceptable level of variation reached. Laboratory instruments were calibrated to guarantee reliability test result and laboratory tests were done according to manufacturer's instructions, with their respective controls.

Ethics approval and consent to participate

Ethical approval was obtained from the ethics committee of Mizan Tepi University research directorate (Ref No: MTU/27/514/44/10). Permission of Sheka Zone, Southwest Ethiopia Health office and the head of the woreda were guaranteed. After explaining the objectives of the study, written consent was obtained from each study participant. For the participants who were not able to read and write, a fingerprint was used as a signature. The referral was considered for study participants with CKD and other medical risk factors.

Results

Socio-demographic characteristics

A total of 326 participants which gives a 95.9% response rate were included in this study. Most participants were within the age category of 18-39 (241; 73.9%) years. The proportions of female participants (59.8%) were relatively higher than male participants (40.2%). Significant differences in age, marital status and occupation were observed between male and females ($p<0.05$) as indicated in Table 1.

Table 1. Socio-economic and demographic characteristics of study participants in Sheka zone, Southwest Ethiopia, 2018.

**Characteristics**	**Overall**	**Men**	**Women**	**p-value**
**Age (**Mean=33.90 ± 11.2 years)				0.011
18–39 years old	241(73.9)	87(66.4)	154(79.0)	
≥ 40 years old	85(26.1)	44(33.6)	41(21.0)	
**Education status**				0.163

Not attended formal education	53(16.3)	17(13.0)	36(18.5)	
Primary (1-8)	116(35.6)	42(32.1)	74(37.9)	
Secondary (9-12)	90(27.6)	44(33.6)	46(23.6)	
Above (12+)	67(20.6)	28(21.4)	39(20.0)	
**Marital status**				0.032
Married	267(81.9)	100(76.3)	167(85.6)	
Others*	59(18.1)	31(23.7)	28(14.4)	
**Religion**				0.477
Christian	242(74.2)	100(41.3)	31(36.9)	
Muslim	84(25.8)	142(58.7)	53(63.1)	
**Occupation**				<0.001
Employed**	53(16.3)	22(16.8)	31(15.9)	
Farming	56(17.2)	37(28.2)	19(9.7)	
Merchant	49(15.0)	36(27.5)	13(6.7)	
Housewife	104(31.9)	3(2.3)	101(51.8)	
Students	42(12.9)	18(13.7)	24(12.3)	
Others***	22(6.7)	15(11.5)	7(3.6)	
**Monthly income ((**Median 2000(IQR= 2200 EB))				0 .110
<2000EB	113(34.7)	38(36.9)	75(46.9)	
≥2000EB	150(46.0)	65(63.1)	85(53.1)	
**Main source of water for drinking**				0.771
Pip water	171(52.5)	70(53.4)	101(51.8)	
Wall/spring	155(47.5)	61(46.6)	94(48.2)	

EB =Ethiopian birr*Single, divorced or widowed ** Private and government employed ***house maid, retired, unemployed

Behavioral and Lifestyle characteristics

Majority of participants didn't engage in vigorous activity/exercise (83.1%) or moderate activity/exercise (72.4%). A very small proportion (3.1%) of participants ever used herbal medicines. About 43 (13.2%) of the participants reported that they used nonsteroidal anti-inflammatory drugs. Nearly one-third of the participants (34.0%) reported that they drink <2 liters of water per day. A significant difference in alcohol and cigarette consumption, moderate exercise/activity, vigorous exercise/activity, and consumption of canned food between males and female were observed as described in Table 2.

Table 2. Behavioral related characteristics of study participants in Sheka zone, Southwest Ethiopia, 2018.

Characteristics	Overall	Men	Women	P-value
**Ever drink alcohol**				<0.001
Yes	46(14.1)	34(26.0)	12(6.2)	
No	280(85.9)	97(74.0)	183(93.8)	
**Ever smoke cigarette**				
Yes	17(5.2)	17(13.0)	0(0.0)	
No	309(94.8)	114(87.0)	195(100.0)	
**Smoking year**				0.098
<10 years	13(76.5)	49(37.4)	91(46.7)	
≥10 years	4(23.5)	82(62.6)	104(53.3)	
**Herbal medicine intake**				0.326 #
Yes	10(3.1)	2(1.5%)	8(4.1)	
No	316(96.9)	129(98.5)	187(95.9)	
**Vigorous exercise/activity**				0.037
Yes	55(16.9)	29(22.1)	26(13.3)	
No	271(83.1)	102(77.9)	169(86.7)	
**Moderate exercise/activity**				0.013
Yes	90(27.6)	46(35.1)	44(22.6)	
No	236(72.4)	85(64.9)	151(77.4)	
**Added salt to food**				0.931
Never	18(5.5)	8(6.1)	10(5.1)	
Occasionally	55(16.9)	22(16.8)	33(16.9)	
With each meal	253(77.6)	101(77.1)	152(77.9)	
**Eat canned/processed food**				<0.001
Never	193(59.2)	63(48.1)	130(66.7)	
Yes (occasionally and often)	133(40.8)	68(51.9)	65(33.3)	
**Daily water intake**				
<2 letters per day	111(34.0)	46(35.1)	65(33.3)	0.739
≥ 2 letters per day	215(66.0)	85(64.9)	130(66.7)	
**Use of over NSAIDs drugs regularly**				0.002
Yes	43(13.2)	8(6.1)	35(17.9)	
No	283(86.8)	123(93.9)	160(82.1)	

Fishers exact test was used

Clinical characteristics/Co-morbidities

One fifth (21.1%; n=71) of the participants were hypertensive: defined as being on antihypertensive drug or having a systolic blood pressure of ≥140mmHg and /or diastolic blood pressure of ≥ 90mmHg. Similarly, one-fifth of the participants (20.2%; n=66) reported history of anemia. However, small proportion of the participants (3.4%; n=11) were diabetic defined as being on drugs for diabetes and/or having blood glucose level >200mg/dl. Overall, there is a significant difference in the proportion of self-reported history of anemia and kidney stone between men and females ($p<0.05$) as described in Table 3.

Table 3. Clinical/co-morbidity related characteristics of study participants in Sheka zone, Southwest Ethiopia, 2018.

Characteristics	Overall	Men	Women	P-value
**Hypertension**				0.215
Yes	71(21.8)	24(18.3)	47(24.1)	
No	255(78.2)	107(81.7)	148(75.9)	
**Diabetes**				0.761#
Yes	11(3.4)	5(3.8)	6(3.1)	
No	315(96.6)	126(96.2)	189(96.9)	
**Anemia**				<0.001
Yes	66(20.2)	6(4.6)	60(30.8)	
No	260(79.8)	125(95.4)	135(69.2)	
**Heart Disease***				0.374 #
Yes	12(3.7)	3(2.3)	9(4.6)	
No	314(96.3)	128(97.7)	186(95.4)	
**Kidney stone**				0.049
Yes	34(10.4)	19(14.5)	15(7.7)	
No	292(89.6)	112(85.5)	180(92.3)	

* Heart Disease includes coronary disease (vessel surgery), heart failure, or heart attack or stork, # Fishers exact test was used

Anthropometric and laboratory measurements

Table 4 describes the mean differences of the anthropometric measurements and laboratory tests between men and women which were assessed using an independent t-test. A significant mean difference in height, BMI, serum creatinine, and eGFR using CKD-EPI and CG were observed between men and women ($p<0.05$). As expected, serum creatinine was higher in men than women ($p < 0.001$) but they had a significantly lower mean estimated creatinine clearance. We also found significantly high level of obesity among women as compared to men ($p<0.001$).

Table 4. Anthropometric and laboratory measurement results of study participants in Sheka zone, Southwest Ethiopia, 2018.

Characteristics	Overall	Men	Women	P-value
**Mean weight (Kg.)**	60.96±9.63	61.49± 9.13	60.61±9.96	0.414
**Mean height(meter)**	1.60± 0.08	1.66±0.08	1.57±0.06	<0.001
**Mean SBP, mmHg (SD)**	121.01±15.1	121.14±12.5	120.92±16.6	0.896
**Mean DBP, mmHg (SD)**	80.11± 10.7	79.30±9.2	80.66±11.6	0.262
**Mean BMI, kg/m2 (SD)**	23.84±4.10	22.52±3.7	24.73±4.1	<0.001
**Mean serum creatinine, mg/dl (SD)**	0.74±0.22	0.99±.31	0.69±.14	<0.001
**Mean creatinine clearance, ml/min (SD)**				
MDRD	131.29±32.5	114.79±37.6	135.03±30.5	0.075
CG	110.67±33.0	90.20±32.6	115.32±32.7	0.033
CKD-EPI	124.34±23.8	107.10±33.3	128.26±19.5	0.010
**Obesity**				<0.001
Yes	25(7.7)	2(1.5)	23(11.8)	
No	301(92.3)	129(98.5)	172(88.2)	

Staging of kidney function and prevalence of chronic kidney disease

Using CKD-EPI estimator, about 23 (7.05 %) of the participants had stage 1 and stage 2 CKD. Whereas, only 1 (0.3 %) of them developed chronic renal failure (stage 3a-5). The overall prevalence of CKD defined as proteinuria of ≥+1 and/or GFR<60 was 7.4 % ((95% confidence interval (CI) [4.6-10.4]).

Using CG- estimator, about 21 (6.4%) of the participants had stage 1 and stage 2 CKD. Whereas, only 5 (1.5%) of them developed chronic renal failure (stage 3a-5). The overall prevalence of CKD using CG-estimator and defined as proteinuria of ≥+1 and/or GFR <60 was 8.0% ((95% confidence interval (CI) [5.2-11.0]). whereas, using MDRD- estimator, about 23 (7.0 %) of the participants had stage 1 and 2 CKD. Only 0.3% of them developed chronic renal failure (stage 3a-5). The overall prevalence of CKD Using MDRD-estimator and defined as proteinuria of ≥+1and/or GFR<60 was 7.4 % ((95% confidence interval (CI) [4.6-10.1]) as showed in Table 5.

Table 5. Stages of kidney functions using the three eGFR estimators in Sheka zone, Southwest Ethiopia, 2018.

Stages	GFR estimation	CKD-Epi n (%)	CG n (%)	MDRD n (%)
1	≥ 90, with proteinuria (≥+1)	19(5.8)	17(5.2)	20(6.1)
2	60–89, with proteinuria (≥+1)	4(1.2)	4(1.2)	3(0.9)
3a	45–59.9, with or without proteinuria (≥+1)	1(0.3)	4(1.2)	1(0.3)
3b	30–44.9, with or without proteinuria (≥+1)	0(0)	1(0.3)	0(0)
4	15–29.9, with or without proteinuria (≥+1)	0(0)	0(0)	0(0)
5	< 15, with or without proteinuria (≥+1)	0(0)	0(0)	0(0)
	Overall CKD	24(7.4)	26(8.0)	24(7.4)

Bivariate and multivariable analysis of associated factors

As the outcome variable is dictums, we applied logistic regression model. Before applying the model, the assumptions of logistic regression were checked. The linear relationship between continuous independent variables (age and income) and their logit transformation were checked. We found that there was no linear relationship between age and income and their log

transformations. As a result, we included age and income as categorical variables in the model. We also investigated the multicollinearity between independent variables using the variance inflation factor. A significant multicollinearity was not identified. We used three separate logistic regression models for three common GFR estimators. However, the models explained only up 41% (Nagelkerke R^2) of the variance in the CKD. The Hosmer & Lemeshow test of the three models was not significant (p>0.05) which suggests good model fitness.

Using all the three GFR estimators, age, hypertension, heart disease, central obesity and use of over the counter NSAIDs drugs regularly were significantly associated with CKD in the Bivariate analysis (p<0.05). In the full adjusted model, only hypertension was significantly associated with CKD using CKD-EPI & MDRD- estimators. On the other hand, age was significantly associated with CKD using CG- estimator in the full adjusted model. Using CKD-EPI & MDRD- estimators, participants who had hypertension were nearly 2.61 times more likely to have CKD than those who didn't have hypertension (p<0.001). Using CG- estimator, participants whose age ≥40 were 3.187 times more likely to exhibit CKD than whose age was 18-39 years (p=0.005). However, central obesity and age showed borderline significant using CKD-EPI & MDRD estimators described in Tables 6-8.

Table 6. Factors associated with CKD in age and sex adjusted logistic regressions using CKD-EPI estimator in Sheka Zone, Southwest Ethiopia, 2018.

**Variables**	**Chronic Kidney Disease**		**COR with 95% CI**	**P value**	**AOR with 95% CI**
**Socio-demographic characteristics**	**Yes (n, %)**	**No (n, %)**			
**Sex**					
Male	6(25.0)	125(41.4)	1		1
Female	18(75.0)	177(58.6)	2.12(0.82-5.49)	0.122	1.031(0.295-3.598)
**Age**					
18-39	13(54.2)	228(75.5)	1		

≥40	11(45.8)	74(24.5)	2.607(1.120-6.067)	0.026	2.347(0.902-6.109)
**Education status**					
Not attended formal education	1(4.2)	52(17.2)	0.642(0.277-1.492)	0.303	
Attended formal education	23(95.8)	250(82.8)	1		
**Marital status**					
Married	21(87.5)	246(81.5)	1		
Others	3(12.5)	56(18.5)	0.628(0.181-2.177)	0.463	
**Religion**					
Christian	17(70.8)	225(74.5)	1		
Muslim	7(29.2)	77(25.5)	1.203(0.481-3.012)	0.693	
**Occupation**					
Employed*	4(7.5)	49(92.5)			
Farming	2(3.6)	54(96.4)	0.454(0.080-2.587)	0.374	
Merchant	4(8.2)	45(91.8)	1.089(0.257-4.613)		
Housewife	11(10.6)	93(89.4)	1.449(0.438-4.789)		
Students	2(4.8)	40(95.2)	0.613(0.107-3.518)		
Others**	1(4.5)	21(95.5)	0.583(0.061-5.535)		
**Ethnicity**					
Sheka (natives)	9(37.5)	77(25.5)	1.753(0.737-4.168)	0.204	
Others***	15(62.5)	225(74.5)	1		
**Monthly income**					
<2000EB	9(45.0)	104(42.8)	1		
≥2000EB	11(55.0)	139(57.2)	0.914(0.366-2.287)	0.848	
**Main source of water for drinking**					
Wall/spring	11(45.8)	144(47.7)	0.928(0.403-2.138)	0.861	
Pip water	13(54.2)	158(52.3)	1		
**Lifestyle/behavioral characteristics**					
**Ever drink alcohol**					

Yes	1(4.2)	45(14.9)	0.248(0.033-1.885)	0.178	0.269(0.033-2.176)
No	23(95.8)	257(85.1)	1		
**Ever smoke cigarette**					
Yes	1(4.2)	16<br>5.3%	0.777(0.099-6.125)	0.811	
No	23(95.8)	286(94.7)	1		
**Smoking year**					
<10 years	0(0.0)	13(81.3)	≅0	.999	
≥10 years	1(100.0)	3(18.8)	1		
**Herbal supplement/ medicine**					
Yes	2(8.3)	8(2.6)	3.341(0.669-16.696)	0.142	0.997(0.150-6.621
No	22(91.7)	294(97.4)	1		
**Use of over the counter NSAIDs drugs regularly**					
Yes	7(29.2)	36(11.9)	3.042(1.181-7.840)	0.021	2.219(0.760-6.483
No	17(70.8)	266(88.1)	1		
**Vigorous exercise /activity**					
Yes	2(8.3)	53(17.5)	0.427(0.097-1.872	0.259	
No	22(91.7)	249(82.5)	1		
**Moderate exercise/activity**					
Yes	9(37.5)	81(26.8)	1.637(0.689-3.887)	0.264	
No	15(62.5)	221(73.2)	1		
**Eat canned/processed foods**					
Never	13(54.2)	180(59.6)	1		
Yes (occasionally and often)	11(45.8)	122(40.4)	1.248(0.542-2.878)	0.603	
**Added salt to food**					
Never	1(4.2)	17(5.6)	0.871(0.109-6.970)		0.878(0.096-8.007)
Occasionally	7(29.2)	48(15.9)	2.160(0.843-5.534)	0.109	1.593(0.546-4.647
With each meal	16(66.7)	237(78.5)	1		
**Daily water intake**					
<2 letters per day	11(45.8)	100(33.1)	1.709(0.739-3.951)	0.210	
≥ 2 letters per day	13(54.2)	202(66.9)	1		
**Co-morbidities/clinical characteristics**					

**Hypertension**					
Yes	12(50.0)	59(19.5)	4.119(1.762-9.629)	0.001	2.614(1.016-6.727
No	12(50.0)	243(80.5)	1		
**Diabetes mellitus**					
Yes	1(4.2)	10(3.3)	1.270(0.156-10.357)	0.824	
No	23(95.8)	292(96.7)	1		
**Anemia**					
Yes	6(25.0)	60(19.9)	1.344(0.512-3.533	0.548	
No	18(75.0)	242(80.1)	1		
**Heart Disease***					
Yes	3(12.5)	9(3.0)	4.651(1.170-18.479)	0.029	2.539(0.456-14.142
No	21(87.5)	293(97.0)	1		
**Kidney stone**					
Yes	4(16.7)	30(9.9)	1.813(0.581-5.657)	0.305	
No	20(83.3)	272(90.1)	1		
**Central obesity**					
Yes	13(54.2)	69(22.8)	3.991(1.711-9.306)	0.001	2.352(0.770-7.188
No	11(45.8)	233(77.2)	1		
**Nutritional status based on BMI**					
Under weight	0(0.0)	26(8.8)	≅0		
Normal weight	15(62.5)	175(58.9)	0.986(0.212-4.589)		
Overweight	7(29.2)	73(24.6)	1.103(0.214-5.684)	0.907	
Obese	2(8.3)	23(7.7)	1		

Table 7. Factors associated with CKD in age and sex adjusted logistic regressions using MDRD estimator in Sheka Zone, Southwest Ethiopia, 2018.

**Variables**	**Chronic Kidney Disease**		**COR with 95% CI**	**P value**	**AOR with 95% CI**
**Socio-demographic characteristics**	**Yes (n, %)**	**No (n, %)**			
**Sex**					
Male	6(25.0)	125(41.4)	1		1
Female	18(75.0)	177(58.6)	2.119(0.818-5.489)	0.122	1.031(0.295-3.598)

**Age**					
18-39	13(54.2)	228(75.5)	1		
≥40	11(45.8)	74(24.5)	2.607(1.120-6.067)	0.026	2.347(0.902-6.109)
**Education status**					
Not attended formal education	1(4.2)	52(17.2)	0.209(0.028-1.582)	0.303	
Attended formal education	23(95.8)	250(82.8)	1		
**Marital status**					
Married	21(87.5)	246(81.5)	1		
Others	3(12.5)	56(18.5)	0.628(0.181-2.177)	0.463	
**Religion**					
Christian	17(70.8)	225(74.5)	1		
Muslim	7(29.2)	77(25.5)	1.203(0.481-3.012)	0.693	
**Occupation**					
Employed*	4(16.7)	49(16.2)			
Farming	2(8.3)	54(17.9)	0.454(0.080-2.587)	0.374	
Merchant	4(16.7)	45(14.9)	1.089(0.257-4.613)		
Housewife	11(45.8)	93(30.8)	1.449(0.438-4.789)		
Students	2(8.3)	40(13.2)	0.613(0.107-3.518)		
Others**	1(4.2)	21(7.0)	0.583(0.061-5.535)		
**Ethnicity**					
Sheka(natives)	9(37.5)	77(25.5)	1.753(0.737-4.168)	0.204	
Others***	15(62.5)	225(74.5)	1		1
**Monthly income**					
<2000EB	9(45.0)	104(42.8)	1		1
≥2000EB	11(55.0)	139(57.2)	0.914(0.366-2.287)	0.848	
**Main source of water for drinking**					
Wall/spring	11(45.8)	144(47.7)	0.928(0.403-2.138)	0.861	
Pip water	13(54.2)	158(52.3)	1		1
**Lifestyle/behavioral characteristics**					
**Ever drink alcohol**					
Yes	1(4.2)	45(14.9)	0.248(0.033-1.885)	0.178	0.269(0.033-2.176)
No	23(95.8)	257(85.1)	1		1
**Ever smoke cigarette**					

Yes	1(4.2)	16(5.3)	0.777(0.099-6.125)	0.811	
No	23(95.8)	286(94.7)	1		
**Smoking year**					
<10 years	0(0.0)	13(81.3)	≅0	0.999	
≥10 years	1(100.0)	3(18.8)	1		
**Herbal supplements medicine**					
Yes	2(8.3)	8(2.6)	3.341(0.669-16.696)	0.142	0.997(0.150-6.621)
No	22(91.7)	294(97.4)	1		1
**Use of over the counter NSAIDs drugs regularly**					
Yes	7(29.2)	36(11.9)	3.042(1.181-7.840)	0.021	2.219(0.760-6.483)
No	17(70.8)	266(88.1)	1		1
**Vigorous exercise /activity**					
Yes	2(8.3)	53(17.5)	0.427(0.097-1.872)	0.259	
No	22(91.7)	249(82.5)	1		
**Moderate exercise/activity**					
Yes	9(37.5)	81(26.8)	1.637(0.689-3.887)	0.264	
No	15(62.5)	221(73.2)	1		
**Eat canned/processed foods**					
Never	13(54.2)	180(59.6)	1		
Yes (occasionally and often)	11(45.8)	122(40.4)	1.248(0.542-2.878)	0.603	
**Added salt to food**					
Never	1(4.2)	17(5.6)	0.871(0.109-6.970)		0.878(0.096-8.007)
Occasionally	7(29.2)	48(15.9)	2.160(0.843-5.534)	0.109	1.593(0.546-4.647)
With each meal	16(66.7)	237(78.5)	1		1
**Daily water intake**					
<2 letters per day	11(45.8)	100(33.1)	1.709(0.739-3.951)	0.210	
≥ 2 letters per day	13(54.2)	202(66.9)	1		
**Co-morbidities/clinical characteristics**					
**Hypertension**					
Yes	12(50.0)	59(19.5)	4.119(1.762-9.629)	0.001	2.614(1.016-6.727)
No	12(50.0)	243(80.5)	1		1
**Diabetes**					
Yes	1(4.2)	10(3.3)	1.270(0.156-10.357)	0.824	
No	23(95.8)	292(96.7)	1		
**Anemia**					

Yes	6(25.0)	60(19.9)	1.344(0.512-3.533)	0.548	
No	18(75.0)	242(80.1)	1		
**Heart Disease***					
Yes	3(12.5)	9(3.0)	4.651(1.170-18.479)	0.029	2.539(0.456-14.142)
No	21(87.5)	293(97.0)	1		1
**Kidney stone**					
Yes	4(16.7)	30(9.9)	1.813(0.581-5.657)	0.305	
No	20(83.3)	272(90.1)	1		
**Central Obesity**					
Yes(obese)	13(54.2)	69(22.8)	3.991(1.711-9.306)	0.001	2.352(0.770-7.188)
No (not obese)	11(45.8)	233(77.2)	1		1
**Nutritional status based on BMI**					
Under weight	0(0.0)	26(8.8)	≅0		
Normal weight	15(62.5)	175(58.9)	0.986(0.212-4.589)		
Overweight	7(29.2)	73(24.6)	1.103(0.214-5.684)	0.907	
Obese	2(8.3)	23(7.7)	1		

Table 8. Factors associated with CKD in age and sex adjusted logistic regressions using CG estimator in Sheka Zone, Southwest Ethiopia, 2018.

**Variables**	**Chronic Kidney Disease**		**Unadjusted**	**P-value**	**Adjusted**
**Socio-demographic characteristics**	**Yes (n, %)**	**No n, %)**			
**Sex**					
Male	6(23.1)	125(41.7)	1		1
Female	20(76.9)	175(58.3)	2.381(0.929-6.100)	0.071	1.340(0.404-4.442)
**Age**					
18-39	13(50.0)	228(76.0)	1		
≥40	13(50.0)	72(24.0)	3.167(1.404-7.141)	0.005	3.187(1.281-7.926)
**Education status**					
Not attended formal education	2(7.7)	51(17.0)	0.407(0.093-1.776)	0.232	
Attended formal education	24(92.3)	249(83.0)	1		1
**Marital status**					
Married	23(88.5)	244(81.3)	1		1

Others	3(11.5)	56(18.7)	0.568(0.165-1.959)	0.371	
**Religion**					
Christian	18(69.2)	224(74.7)	1		1
Muslim	8(30.8)	76(25.3)	1.310(0.547-3.135)	0.544	
**Occupation**					
Employed*	4(15.4)	49(16.3)	1		
Farming	3(11.5)	53(17.7)	0.693(0.148-3.256)		
Merchant	4(15.4)	45(15.0)	1.089(0.257-4.613)	0.438	
Housewife	12(46.2)	92(30.7)	1.598(0.489-5.218)		
Students	2(7.7)	40(13.3)	0.613(0.107-3.518)		
Others**	1(3.8)	21(7.0)	0.583(0.061-5.535)		
**Ethnicity**					
Sheka (natives)	10(38.5)	76(25.3)	1.842(0.802-4.232)	0.150	
Others***	16(61.5)	224(74.7)	1		1
**Monthly income**					
<2000EB	11(50.0)	102(42.3)	1		1
≥2000EB	11(50.0)	139(57.7)	0.734(0.306-1.758)	0.488	
**Main source of water for drinking**					
Wall/spring	13(50.0)	142(47.3)	1.113(0.499-2.480)	0.794	
Pip water	13(50.0)	158(52.7)	1		1
**Lifestyle/behavioral characteristics**					
**Ever drink alcohol**					
Yes	1(3.8)	45(15.0)	0.227(0.030-1.715)	0.151	0.253(0.032-2.025)
No	25(96.2)	255(85.0)	1		
**Ever smoke cigarette**					
Yes	1(3.8)	16(5.3)	0.710(0.090-5.578)	0.745	
No	25(96.2)	284(94.7)	1		
**Smoking year**					
<10 years	0(0.0)	13(81.3)	≅0	0.999	
≥10 years	1(100.0)	3(18.8)	1		
**Herbal supplements /medicine**					
Yes	2(7.7)	8(2.7)	3.042(0.611-15.132)	0.174	1.106(0.167-7.342)
No	24(92.3)	292(97.3)	1		

**Use of over the counter NSAIDs drugs regularly**					
Yes	7(26.9)	36(12.0)	2.702(1.062-6.875)	0.037	1.937(0.673-5.576)
No	19(73.1)	264(88.0)	1		
**Vigorous exercise /activity**					
Yes	2(7.7)	53(17.7)	0.388(0.089-1.694)	0.208	
No	24(92.3)	247(82.3)	1		
**Moderate exercise/activity**					
Yes	10(38.5)	80(26.7)	1.719(0.749-3.944)	0.201	
No	16(61.5)	220(73.3)	1		
**Eat canned/processed foods**					
Never	15(57.7)	178(59.3)	1		
Yes (occasionally and often)	11(42.3)	122(40.7)	1.070(0.475-2.409)	0.870	
**Added salt to food**					
Never	1(3.8)	17(5.7)	0.768(0.097-6.104)		0.716(0.078-6.543)
Occasionally	7(26.9)	48(16.0)	1.904(0.754-4.809)	0.173	1.471(0.511-4.233)
With each meal	18(69.2)	235(78.3)	1		
**Daily water intake**					
<2 letters per day	11(42.3)	100(33.3)	1.467(0.650-3.311)	0.357	
≥ 2 letters per day	15(57.7)	200(66.7)	1		
**Co-morbidities/clinical**					
**Hypertension**					
Yes	12(46.2)	59(19.7)	3.501(1.539-7.965)	0.003	2.105(0.836-5.299)
No	14(53.8)	241(80.3)	1		
**Diabetes**					
Yes	1(3.8)	10(3.3)	1.160(0.143-9.433)	0.890	
No	25(96.2)	290(96.7)	1		
**Anemia**					
Yes	6(23.1)	60(20.0)	1.200(0.462-3.119)	0.708	
No	20(76.9)	240(80.0)	1		
**Heart Disease***					
Yes	3(11.5)	9(3.0)	4.217(1.068-16.661)	0.040	2.555(0.462-14.133)
No	23(88.5)	291(97.0)	1		
**Kidney stone**					
Yes	4(15.4)	30(10.0)	1.636(0.529-5.066)	0.393	
No	22(84.6)	270(90.0)	1		
**Central Obesity**					

Yes	14(53.8)	68(22.7)	3.980(1.758-9.011)	0.001	2.147(0.748-6.162)
No	12(46.2)	232(77.3)	1		
**Nutritional status based on BMI**					
Under weight	1(3.8)	25(8.5)	0.460(0.039-5.418)	0.537	
Normal weight	15(57.7)	175(59.3)	0.986(0.212-4.589)		
Overweight	8(30.8)	72(24.4)	1.278(0.253-6.451)		
Obese	2(7.7)	23(7.8)	1		

Discussion

In the current study, we assessed the prevalence and associated risk factors of chronic kidney diseases among the urban adult population in Sheka zone using the three commonest estimators of kidney functions. Over 7% of urban adults had CKD regardless of whether CKD-EPI, CG or MDRD GFR estimators were used. This finding is comparable with the prevalence of CKD reported in other sub-Saharan African counties; 10.4% in Nigeria [14] , 12.4% in Kinshasa Congo [15], 10% in Cameroon [10], and 7% in Tanzania with 15.2% in the urban population[16]. The prevalence of CKD was slightly higher using the CG GFR estimator. It has been documented that CG overestimates the true prevalence of CKD[10,16]. But, a similar prevalence of CKD was estimated using CKD-EPI and MDRD estimators. This may indicate that either CKD-EPI or MDRD can be used to estimate GFR in this study area. Other study findings, for instance, in Southern Africa and Ghana also showed that there is a high agreement between these two GFR estimators [17,18].

Using NKF/KDOQI staging, we found that most of the participants were in stage I & II. The finding is in line with several study findings in developing countries [9-11]. People within the early stage of CKD may not aware of their status. In fact, several studies showed that the majority of

CKD patients don't aware their kidney problems [15,19,20]. Identification of people in the early stage of CKD is important. This is because the cardiovascular risks associated with stage I & II is nearly equal to that of stage III [21].

After adjusting socio-demographic, lifestyle and co-morbidities related variables, only hypertension and advanced age were found to be significantly associated with CKD using CKD-EPI/MDRD & CG estimators respectively. However, more of the increased prevalence of CKD in this study area seems to be unexplained. In line with our study finding, a recent study finding in Tanzania and Uganda showed that the majority of the increase in the risk of CKD was not explained [9,11]. Unexplained risk factors of CKD in this study area may suggest that other non-traditional risk factors might contribute to the high burden of CKD. Some study findings in another part of the world showed that nontraditional risk factors such as heavy metals and agro-chemicals may contribute to CKD in tropical and semi-tropical areas [22,23]. Some other studies suggested that infectious diseases such as HIV/AIDS, Schistosomiasis and Leishmaniasis are also associated with CKD [24-26]. However, as a limitation of this study, we didn't control the confounding effect of these risk factors of CKD. Large-scale epidemiological studies are needed to better understand the specific risk factors of CKD and, to examine the potential but unmeasured above risk factors.

As documented in the broad literature [7,10,11,19], advanced age is a well-known risk factor of CKD. As age increases, there may be salt sensitization, hardening of arteries and scarring of the tiny blood vessels in the kidney. This may, in turn, enhance protein excretion and decrease GFR [27-29]. However, as many of the adult participants in the current study didn't have a birth certificate, they may underestimate their age. Underestimation of age could lead to overestimation of GFR using any of the methods used to calculate GFR. This may in turn underestimate the true prevalence of CKD in this study area.

In the current study, among the traditional risk factors of CKD, hypertension was found to be significantly associated with CKD. This is in line with several community-based study findings [10,11,14,20]. Hypertension may elevate intraglomerular pressure which results in glomerulosclerosis and eventual protein trafficking [30,31]. However, as a limitation of this study (chicken egg dilemma), it is hard to distinguish whether hypertension causes CKD or CKD causes hypertension. An increased risk of CKD among hypertensive participants may warrant urban adults to adhere to treatment and control their blood pressures. Studies have shown that interventions that decrease blood pressure levels in patients with proteinuria delay progression to CKD [32,33].

Co-morbidity related factors such as diabetes, anemia, kidney stone; heart disease, central obesity and nutritional status measured by BMI were not significantly associated with CKD. However, central obesity showed a borderline significant. It has been documented that waist circumference or waist to hip ratio predicts cardiovascular risks than BMI [34]. The low prevalence of the above-mentioned co-morbidities in our participants makes any comment of their association difficult to ascertain.

In the current study, behavioral factors such as alcohol intake and cigarette smoking were not significantly associated with CKD. This is in line with several study findings [14,35,36]. Alcohol intake and cigarette smoking are often considered anti-social habits in Ethiopia so people may give a negative response. This may, in turn, nullify the true association between alcohol intake, cigarette smoking and, CKD.

Overall, in this study area, CKD is significantly associated with age ≥40 years old and hypertension. Early screening of high-risk populations (elderly and hypertensive peoples in the case of this study area) may help to diagnose people at the early stage of CKD. This is very

important as Ethiopian health care institutions may not be able to support the economic burden of providing dialysis and renal replacement therapy to all patients. Dialysis and renal replacement therapy are not free in Ethiopia. This may result catastrophic out of the pocket expenditure. Thus, early screening of high-risk populations to CKD is important to minimize complications and out of pocket expenditures.

This study was the first community-based assessment of CKD in Southwest Ethiopia using the three common GFR estimators. The finding of the study will provide information about the urban prevalence of CKD in Sheka zone. However, this study has several limitations. The cross-sectional nature of this study restricted us to infer causality. The common GFR measures used in this study were not validated in Ethiopia. The absence of reliable and validated measures of kidney function tests has made difficult the estimation of CKD in Africa [16,18]. This study exclusively focused on the urban population. Thus, the estimated CKD may not reflect the prevalence and risk factors of rural communities. Because of security and financial restrictions, we didn't make a three months control of positive findings as suggested by the KDIGO guideline. Some risk factors identified elsewhere such as heavy metals, agrochemicals, Schistosomiasis, Leishmaniasis and HIV [22-26] were not investigated in this study.

Conclusions

The prevalence of CKD was comparable with other sub-Saharan African countries. Only hypertension and age ≥ years old were significantly associated with CKD. More of the increased prevalence of CKD in the current study remained unexplained and deserves further study. Policymakers and programmers need to design strategies and encourage high-risk populations to be screened as early as possible. It is better if clinicians should also consider kidney function marker tests for hypertensive and patients older than 40 years old.

Acknowledgments

Special gratitude goes to Mizan-Tepi University for providing funds for this study. The authors would like to acknowledge all the data collectors, supervisors and study participants.

References

1. Jha V, Garcia-Garcia G, Iseki K, Li Z, Naicker S, et al. (2013) Chronic kidney disease: global dimension and perspectives. The Lancet 382: 260-272.

2. Hill NR, Fatoba ST, Oke JL, Hirst JA, O'Callaghan CA, et al. (2016) Global prevalence of chronic kidney disease–a systematic review and meta-analysis. PloS one 11.

3. Yirsaw BD (2012) Letter to the Editor-Chronic kidney disease in sub-Saharan Africa: Hypothesis for research demand. Annals of African medicine 11: 119-120.

4. Baynes HW, Negash M, Geremew D, Getaneh Z (2019) Chronic Kidney Disease among Human Immunodeficiency Virus Positive Patients on Antiretroviral Therapy in Sub-Saharan Africa: A Systematic Review and Meta-analysis. Saudi Journal of Kidney Diseases and Transplantation 30: 1190-1200.

5. Global HIV and AIDS Statistics. 2018 Global HIV Statistics. Available from: http://www.avert.org/global-hiv-and-aids-statistics. (Accessed on April 11, 2020).

6. World Health Organization. Noncommunicable Dseases Country Profiles 2018. WHO, 2018 (Accessed on April 10, 2020).

7. Fiseha T, Kassim M, Yemane T (2014) Chronic kidney disease and underdiagnosis of renal insufficiency among diabetic patients attending a hospital in Southern Ethiopia. BMC nephrology 15: 198.

8. Mekuria Y, Yilma D, Mekonnen Z, Kassa T, Gedefaw L (2016) Renal Function Impairment and Associated Factors among HAART Naïve and Experienced Adult HIV Positive Individuals in Southwest Ethiopia: A Comparative Cross Sectional Study. PloS one 11.

9. Stanifer JW, Maro V, Egger J, Karia F, Thielman N, et al. (2015) The epidemiology of chronic kidney disease in Northern Tanzania: a population-based survey. PloS one 10.

10. Kaze FF, Halle M-P, Mopa HT, Ashuntantang G, Fouda H, et al. (2015) Prevalence and risk factors of chronic kidney disease in urban adult Cameroonians according to three common estimators of the glomerular filtration rate: a cross-sectional study. BMC nephrology 16: 96.

11. Kalyesubula R, Nankabirwa JI, Ssinabulya I, Siddharthan T, Kayima J, et al. (2017) Kidney disease in Uganda: a community based study. BMC nephrology 18: 116.

12. Coudène P, Marson B, Badiou S, Flavier S, Anelli S, et al. (2005) Evaluation of the ABX Pentra 400: a newly available clinical chemistry analyser. Clinical Chemistry and Laboratory Medicine (CCLM) 43: 782-792.

13. Levey AS, Inker LA (2014) Definition and staging of chronic kidney disease in adults. V: UpToDate, Post TW (Ed), UpToDate, Waltham, MA (Pridobljeno 5 Sep 2014).

14. Afolabi MO, Abioye-Kuteyi AE, Arogundade FA, Bello IS (2009) Prevalence of chronic kidney disease in a Nigerian family practice population. South African Family Practice 51.

15. Sumaili EK, Krzesinski J-M, Zinga CV, Cohen EP, Delanaye P, et al. (2008) Prevalence of chronic kidney disease in Kinshasa: results of a pilot study from the Democratic Republic of Congo. Nephrology Dialysis Transplantation 24: 117-122.

16. Stanifer JW, Jing B, Tolan S, Helmke N, Mukerjee R, et al. (2014) The epidemiology of chronic kidney disease in sub-Saharan Africa: a systematic review and meta-analysis. The Lancet Global Health 2: e174-e181.

17. Matsha TE, Yako YY, Rensburg MA, Hassan MS, Kengne AP, et al. (2013) Chronic kidney diseases in mixed ancestry south African populations: prevalence, determinants and concordance between kidney function estimators. BMC nephrology 14: 75.

18. Eastwood JB, Kerry SM, Plange-Rhule J, Micah FB, Antwi S, et al. (2010) Assessment of GFR by four methods in adults in Ashanti, Ghana: the need for an eGFR equation for lean African populations. Nephrology Dialysis Transplantation 25: 2178-2187.

19. Abu-Aisha H, Elhassan A, Khamis A, Abu-Elmaali A (2009) Chronic kidney disease in police forces households in Khartoum, Sudan: pilot report. Arab Journal of Nephrology and Transplantation 2: 21-26.

20. HWANG SJ, TSAI JC, CHEN HC (2010) Epidemiology, impact and preventive care of chronic kidney disease in Taiwan. Nephrology 15: 3-9.

21. Glassock RJ, Winearls C (2008) Screening for CKD with eGFR: doubts and dangers. Clinical Journal of the American Society of Nephrology 3: 1563-1568.

22. Lozier M, Turcios-Ruiz RM, Noonan G, Ordunez P (2016) Chronic kidney disease of nontraditional etiology in Central America: a provisional epidemiologic case definition for surveillance and epidemiologic studies. Revista Panamericana de Salud Pública 40: 294-300.

23. Chapman E, Haby MM, Illanes E, Sanchez-Viamonte J, Elias V, et al. (2019) Risk factors for chronic kidney disease of non-traditional causes: a systematic review.

24. Wyatt CM, Meliambro K, Klotman PE (2012) Recent progress in HIV-associated nephropathy. Annual review of medicine 63: 147-159.

25. Wyatt CM, Klotman PE, D'Agati VD. HIV-associated nephropathy: clinical presentation, pathology, and epidemiology in the era of antiretroviral therapy; 2008. Elsevier. pp. 513-522.

26. da Silva Junior GB, Duarte DB, Barros EJG, Daher EDF (2013) Schistosomiasis-associated kidney disease: A review. Asian Pacific Journal of Tropical Disease 3: 79-84.

27. Iseki K, Iseki C, Ikemiya Y, Kinjo K, Takishita S (2007) Risk of developing low glomerular filtration rate or elevated serum creatinine in a screened cohort in Okinawa, Japan. Hypertension research 30: 167.

28. Shannon R, Minaker K, Rowe J (1984) The influence of age on water balance in man. Semin Nephrol 4: 346-352.

29. Poulter N, Khaw K, Hopwood B, Mugambi M, Peart W, et al. (1984) Salt and blood pressure in various populations. Journal of cardiovascular pharmacology 6: S197-203.

30. Grimm Jr RH, Svendsen KH, Kasiske B, Keane WF, Wahl MM (1997) Proteinuria is a risk factor for mortality over 10 years of follow-up. Kidney International Supplement.

31. Bianchi S, Bigazzi R, Campese VM (1999) Microalbuminuria in essential hypertension: significance, pathophysiology, and therapeutic implications. American journal of kidney diseases 34: 973-995.

32. Jafar TH, Stark PC, Schmid CH, Landa M, Maschio G, et al. (2003) Progression of chronic kidney disease: the role of blood pressure control, proteinuria, and angiotensin-converting enzyme inhibition: a patient-level meta-analysis. Annals of internal medicine 139: 244-252.

33. Peterson JC, Adler S, Burkart JM, Greene T, Hebert LA, et al. (1995) Blood pressure control, proteinuria, and the progression of renal disease: the Modification of Diet in Renal Disease Study. Annals of internal medicine 123: 754-762.

34. Taylor EN, Stampfer MJ, Curhan GC (2005) Obesity, weight gain, and the risk of kidney stones. Jama 293: 455-462.

35. Vupputuri S, Sandler DP (2003) Lifestyle risk factors and chronic kidney disease. Annals of epidemiology 13: 712-720.

36. Stengel B, Tarver–Carr ME, Powe NR, Eberhardt MS, Brancati FL (2003) Lifestyle factors, obesity and the risk of chronic kidney disease. Epidemiology 14: 479-487.

Supporting information

S1 File. Questionnaire in English and local language (Amharic).

Ref No: PONE-D-20-08526

Title: Chronic Kidney Disease and Associated Factors among Adult Population in Southwest Ethiopia

Response to reviewers

1. We recommend to include the potential impact of the kebeles in the results of the survey.

   ***Response**: Dear reviewer, all the kebeles included in this study were urban kebeles and assumed to be homogenous. Therefore, the prevalence of CKD is not expected to vary among the included kebeles.*

2. We require to suppress these first lines and start the paper directly with the CKD.

   ***Response**: We have deleted the first paragraph and started the paper directly with the CKD (Introduction section, page 2, line 38-44).*

3. Authors are required to re-define what advanced age means or modify the age ranges. > 40 years cannot be considered advanced age

   ***Response**: Dear reviewer thank you very much for this comment. We couldn't find a standard definition for advanced age for sub-Saharan countries. For this reason, we have removed the term "advanced age" and simply we categorized age as ≥40 years old and <40 years old. The age category was decided for comparison (Compare with other studies) (Abstract section, page 2, line 30; Discussion section, page 25, line 316; Conclusions section, page 26, line 337and 341).*

4. The increased prevalence of CKD in the current study remains unexplained and scarcely discussed. Authors are required to recommend the use of CKD-EPI, MDRD or CG to estimate CKD prevalence in order to make recommendations.

   ***Response**: Even though the aim of this study is not to recommend the use of CKD-EPI, MDRD or CG, we have incorporated recommendations on the use of CKD-EPI, MDRD, or CG to estimate CKD prevalence in this study area (Abstract section, page 2, line 27-28).*

5. The paper is suitable for publication but needs to focus in the novelty and originality of the studied population.

   ***Response**: Dear reviewer, thank you for this comment. At this stage, we can't change the study population. It is difficult to say that the study population is unique. This study was*

conducted among the urban population in the Sheka zone. Therefore, the population may share similar characteristics with other urban populations in Ethiopia. However, this study is the first community-based study in the study area and in Ethiopia at large. In fact, this study has certain limitations. We have included those limitations in the manuscript.

Response to Editor

1. There are similar papers as Sumaili et al in 2009 about prevalence of Chronic Kidney Disease in the Democratic Republic of Congo. Although there is no significant new clinical data, the study is based in a survey and there are some socio-demographic data that are of interest. The survey is based in an administrative Ethiopian distribution of kebeles, making it original. However, these points are not discussed along the paper.

    Response*: Dear editor, all the kebeles included in this study were urban kebeles and assumed to be homogenous. Therefore, the prevalence of CKD is not expected to vary among the included kebeles.*

2. It would be of interest to compare the urban and rural areas.

    Response*: Dear editor, this study was exclusively studied in urban areas. Therefore, it is difficult to compare urban and rural areas.*

3. Moreover, the introduction is initially focused in the global burden of noncommunicable diseases (NCD) but this not the main point along the paper.

    Response*: We have deleted the first paragraph and started the paper directly with the CKD (page 2, line 38-44).*

4. Hypertension and advanced age were significantly associated with the excess risk of CKD, however the advanced age criteria is > 40 years. Authors must re-define what advanced age means or modify the age ranges.

    Response*: Dear editor, thank you very much for this comment. We couldn't find a standard definition for advanced age for sub-Saharan countries. For this reason, we removed the term "advanced age" and simply we categorized age as ≥ 40 years old and <40 years old. The age category was decided for comparison (Compare with other studies) (Abstract section, page 2, line 30; Discussion section, page 25, line 316; Conclusions section, page 26, line 337and 341).*

5. The increased prevalence of CKD in the current study remains unexplained and scarcely discussed. Authors must recommend the use of CKD-EPI, MDRD or CG to estimate CKD prevalence in order to make recommendations.

   ***Response**: Even though the aim of this study is not to recommend the use of CKD-EPI, MDRD, or CG, we have incorporated recommendation on use of CKD-EPI, MDRD or CG to estimate CKD prevalence in this study area (page 2, line 27-28)*

6. The paper is suitable for publication but needs to focus in the novelty and originality of the studied population.

   ***Response**: Dear editor, thank you for this comment. At this stage, we can't change the study population. It is difficult to say that the study population is unique. This study was conducted among the urban population in the Sheka zone. Therefore, the population may share similar characteristics with other urban populations in Ethiopia. However, this study is the first community-based study in the study area and in Ethiopia at large. In fact, this study has certain limitations. We have included those limitations in the manuscript.*

7. I consider that after this correction in the introduction (eliminate the initial lines related to NCD, and start with CKD prevalence and the collected data related with general and CKD populations

   ***Response**: Corrected based on your comment.*

8. If you developed a questionnaire as part of this study and it is not under a copyright more restrictive than CC-BY, please include a copy, in both the original language and English, as Supporting Information.

   ***Response**: We have uploaded a questionnaire both in English and local language (Amharic) as supporting information.*
