# Supplementary material for: Chronic kidney disease and associated factors among adult population in Southwest Ethiopia
Source: PLoS One. 2022 Mar 3;17(3):e0264611. doi: 10.1371/journal.pone.0264611 (PMC8893675; doi:10.1371/journal.pone.0264611)
Supplement: Attachment — Submitted filename: Response to Reviewers.docx [file pone.0264611.s003.docx]

**Ref No:** PONE-D-20-08526 **Title:** Chronic Kidney Disease and Associated Factors among Adult Population in Southwest Ethiopia

**Response to reviewers**

1. We recommend to include the potential impact of the kebeles in the results of the survey.

***Response****: Dear reviewer, all the kebeles included in this study were urban kebeles and assumed to be homogenous. Therefore, the prevalence of CKD is not expected to vary among the included kebeles.*

2. We require to suppress these first lines and start the paper directly with the CKD.

***Response****: We have deleted the first paragraph and started the paper directly with the CKD (Introduction section, page 2, line 38-44).*

3. Authors are required to re-define what advanced age means or modify the age ranges. > 40 years cannot be considered advanced age

***Response****: Dear reviewer thank you very much for this comment. We couldn’t find a standard definition for advanced age for sub-Saharan countries. For this reason, we have removed the term “advanced age” and simply we categorized age as ≥40 years old and <40 years old. The age category was decided for comparison (Compare with other studies) (Abstract section, page 2, line 30; Discussion section, page 25, line 316; Conclusions section, page 26, line 337and 341).*

4. The increased prevalence of CKD in the current study remains unexplained and scarcely discussed. Authors are required to recommend the use of CKD-EPI, MDRD or CG to estimate CKD prevalence in order to make recommendations.

***Response****: Even though the aim of this study is not to recommend the use of CKD-EPI, MDRD or CG, we have incorporated recommendations on the use of CKD-EPI, MDRD, or CG to estimate CKD prevalence in this study area (Abstract section, page 2, line 27-28).*

5. The paper is suitable for publication but needs to focus in the novelty and originality of the studied population.

***Response****: Dear reviewer, thank you for this comment. At this stage, we can’t change the study population. It is difficult to say that the study population is unique. This study was conducted among the urban population in the Sheka zone. Therefore, the population may share similar characteristics with other urban populations in Ethiopia. However, this study is the first community-based study in the study area and in Ethiopia at large. In fact, this study has certain limitations. We have included those limitations in the manuscript.*

**Response to Editor**

1. There are similar papers as Sumaili et al in 2009 about prevalence of Chronic Kidney Disease in the Democratic Republic of Congo. Although there is no significant new clinical data, the study is based in a survey and there are some socio-demographic data that are of interest. The survey is based in an administrative Ethiopian distribution of kebeles, making it original. However, these points are not discussed along the paper.

***Response****: Dear editor, all the kebeles included in this study were urban kebeles and assumed to be homogenous. Therefore, the prevalence of CKD is not expected to vary among the included kebeles.*

2. It would be of interest to compare the urban and rural areas.

***Response****: Dear editor, this study was exclusively studied in urban areas. Therefore, it is difficult to compare urban and rural areas.*

3. Moreover, the introduction is initially focused in the global burden of noncommunicable diseases (NCD) but this not the main point along the paper.

***Response****: We have deleted the first paragraph and started the paper directly with the CKD (page 2, line 38-44).*

4. Hypertension and advanced age were significantly associated with the excess risk of CKD, however the advanced age criteria is > 40 years. Authors must re-define what advanced age means or modify the age ranges.

***Response****: Dear editor, thank you very much for this comment. We couldn’t find a standard definition for advanced age for sub-Saharan countries. For this reason, we removed the term “advanced age” and simply we categorized age as ≥ 40 years old and <40 years old. The age category was decided for comparison (Compare with other studies) (Abstract section, page 2, line 30; Discussion section, page 25, line 316; Conclusions section, page 26, line 337and 341).*

5. The increased prevalence of CKD in the current study remains unexplained and scarcely discussed. Authors must recommend the use of CKD-EPI, MDRD or CG to estimate CKD prevalence in order to make recommendations.

***Response****: Even though the aim of this study is not to recommend the use of CKD-EPI, MDRD, or CG, we have incorporated recommendation on use of CKD-EPI, MDRD or CG to estimate CKD prevalence in this study area (page 2, line 27-28)*

6. The paper is suitable for publication but needs to focus in the novelty and originality of the studied population.

***Response****: Dear editor, thank you for this comment. At this stage, we can’t change the study population. It is difficult to say that the study population is unique. This study was conducted among the urban population in the Sheka zone. Therefore, the population may share similar characteristics with other urban populations in Ethiopia. However, this study is the first community-based study in the study area and in Ethiopia at large. In fact, this study has certain limitations. We have included those limitations in the manuscript.*

7. I consider that after this correction in the introduction (eliminate the initial lines related to NCD, and start with CKD prevalence and the collected data related with general and CKD populations

***Response****: Corrected based on your comment.*

8. If you developed a questionnaire as part of this study and it is not under a copyright more restrictive than CC-BY, please include a copy, in both the original language and English, as Supporting Information.

***Response****: We have uploaded a questionnaire both in English and local language (Amharic) as supporting information.*
