# Supplementary material for: Chronic kidney disease and associated factors among adult population in Southwest Ethiopia
Source: PLoS One. 2022 Mar 3;17(3):e0264611. doi: 10.1371/journal.pone.0264611 (PMC8893675; doi:10.1371/journal.pone.0264611)
Supplement: Attachment — Submitted filename: The Editor PlosOne.docx [file pone.0264611.s005.docx]

The Editor,

PLOS ONE.

Dear Sir,

Review of the Manuscript Titled: “Chronic Kidney Disease and Associated Factors among Adult Population in Southwest Ethiopia” (PONE-D-20-08526R1)

I have reviewed the manuscript and my comments are highlighted in the manuscript.

1. The work is original and will contribute to body of knowledge
2. The author should pay attention and correction the minor edits in the manuscript
3. Figures should carry units
4. Presentation of tables should not be merged together like tables 6-8. Each should be presented separately, highlighting what is significant and relevant to the study.
5. Discussions should focus more on the findings based on the objectives of the study
6. The conclusion should be derived from the outcomes or findings of the study and should not be inferred or extrapolated.

I thank you for giving me the opportunity to review the work.

Kind regards,

**Dr Timothy Olanrewaju**

Global Health Scholar, University Medical Center Utrecht, Utrecht University, Utrecht, The Netherlands.

Consultant Physician and Nephrologist, Department of Medicine University of Ilorin Teaching Hospital, and University of Ilorin, Ilorin, Nigeria.

31st May, 2021
