# Supplementary material for: Chronic kidney disease and associated factors among adult population in Southwest Ethiopia
Source: PLoS One. 2022 Mar 3;17(3):e0264611. doi: 10.1371/journal.pone.0264611 (PMC8893675; doi:10.1371/journal.pone.0264611)
Supplement: Attachment — Submitted filename: Response to Reviewers.docx [file pone.0264611.s006.docx]

**Ref No:** PONE-D-20-08526R1

**Title:** Chronic Kidney Disease and Associated Factors among Adult Population in Southwest Ethiopia

**Response to reviewers**

**Reviewer #1:**

**1. The work is original and will contribute to body of knowledge**

***Response****: Dear reviewer, thanks for your remark.*

**2. The author should pay attention and correction the minor edits in the manuscript**

***Response****: Dear Reviewer, thanks for your comment and we have corrected the minor* *edits within the manuscript accordingly.*

**3. Figures should carry units**

***Response****: We have added the units with figures accordingly (Abstract section, page 2, line 26-27, Result section, page 13, line 217, 229 ).*

**4. Presentation of tables should not be merged together like tables 6-8. Each should be** presented separately, highlighting what is significant and relevant to the study.

***Response****: We have cited the tables separately (Result section, page 14, line 251).*

**5. Discussions should focus more on the findings based on the objectives of the study**

***Response****: Dear reviewer, we appreciate your comment. In the discussion section, we tried to be focused based on the two specific objectives of the study. i. the prevalence of CKD based on the GFR estimators and ii. associated factors of CKD. The discussion is based on the finding of the two objectives.*

**6. The conclusion should be derived from the outcomes or findings of the study and should not be inferred or extrapolated.**

***Response****: Dear reviewer, we appreciate your comment and we have corrected the conclusion section mainly on the conclusion on the prevalence of CKD and avoid extrapolation during the conclusion (Conclusions, page 26, line 339).*

**Reviewer #2:**

**1. On page 13, row 229, please clarify the sentence “As the outcome variable is dictums, we applied logistic regression model”.**

***Response****: Thanks for your comment. The sentence “As the outcome variable is dictums, we applied logistic regression model” is to means “As the outcome variable is dichotomous, we applied logistic regression model.” We re-wrote the sentence. It’s to indicate the outcome variable CKD has one of two possible values and fulfills one of the logistic regression assumptions (Result section, page 14, line 232).*

**2. Please indicate in the methods how was central obesity defined. The methods seem to define obesity only and waist circumference, which is not the same as central obesity. The latter is habitually defined as waist to height ratio.**

***Response****: Thanks for your comment and keen observation. The waist circumference is used to assess central obesity and it’s indicated in previous studies [1, 2]. The waist circumference in the current study is used to define central obesity with the cut-point for male ≥102 cm and female ≥88cm. We have corrected the statement accordingly in the definition of variables section of the manuscript (Methods section, Variables’ definition, page 6, line 132-133).*

*1. Ahmad N, Adam SI, Nawi AM, Hassan MR, Ghazi HF. Abdominal Obesity Indicators: Waist Circumference or Waist-to-hip Ratio in Malaysian Adults Population. Int J Prev Med. 2016 Jun 8;7:82. doi: 10.4103/2008-7802.183654. PMID: 27330688; PMCID: PMC4910307.*

*2. Lean MEJ, Han TS & Morrison CE (1995): Waist circumference as a measure for indicating the need for weight management. Br. Med. J. 311, 158–161.*

**3. There are a couple of places in the results where the statement “developed chronic renal failure” is being used. Since this is a cross-sectional study, time-dependent terminology should be avoided. The authors may wish to use something on the line of “had chronic renal failure” instead.**

***Response****: We appreciate your comment and have used “had chronic renal failure” rather than “developed chronic renal failure” accordingly (Result section, page 12, 13, line 18,222, 225).*

**4. The lack of associations with other risk factors than HT might be due to the low prevalence of these other factors, i.e. CVD, diabetes, in the studied population. The authors may wish to comment on this aspect.**

***Response****: Dear Reviewer, we appreciate your comment. We strongly believe that the lack of association between CKD and factors such as Diabetes and CVD in the current study might be the low prevalence or the low number of study participants with diabetes and CVD that result from no association during logistic regression analysis. Chronic diseases such as diabetes and CVD are known risk factors indicated in different studies.*
