# Supplementary material for: Chronic kidney disease and associated factors among adult population in Southwest Ethiopia
Source: PLoS One. 2022 Mar 3;17(3):e0264611. doi: 10.1371/journal.pone.0264611 (PMC8893675; doi:10.1371/journal.pone.0264611)
Supplement: Attachment — Submitted filename: Response to Reviewers.docx [file pone.0264611.s007.docx]

RESPONSE TO EDITOR AND REVIEWER COMMENTS

Dear Editor and Reviewers,

The authors sincerely appreciate the time and resources invested in giving such a thorough review. Thank you for the previous comments, suggestions and corrections on the manuscript. We have made efforts to address them and revise the manuscript in line with the comments. The changes have been highlighted in red and tracked in the manuscript. We look forward to a favorable and kind re-consideration.

Editor comment

The article is much improved after addressing the reviewers comments. However, I think that it needs further improvement and incorporation of relevant literature. In this connection, I suggest to cite the following very relevant articles either in Introduction or Discussion section of the manuscript:

<https://link.springer.com/article/10.1007/s11845-018-1813-2>

<https://bmcpublichealth.biomedcentral.com/articles/10.1186/s12889-019-6796-z>

<https://link.springer.com/article/10.1007/s11255-018-1834-9>

<https://link.springer.com/article/10.1186/s40545-019-0169-y>

<https://www.hindawi.com/journals/bmri/2016/9710965/>

***Response****: Dear Editor, thank you for your insights and the suggested articles to be incorporated within the manuscript. The articles are very interesting. We have added a reference that supports a statement in the discussion section (Ref#30, Page 25, Line 29). However, the other articles are studies on hemodialysis patients (focuses on depression among hemodialysis patients, hypertension control among hemodialysis patients, management of patient care in hemodialysis, and on factors affecting to achieve dry weight in post-dialysis) and we couldn’t cite as our study mainly focuses on the prevalence of CKD through a community-based cross-sectional study. But we will use it for other studies in the future.*
